# Mechanistic Evaluations of the Effects of Auranofin Triethylphosphine Replacement with a Trimethylphosphite Moiety

**DOI:** 10.1021/acs.inorgchem.3c01280

**Published:** 2023-06-21

**Authors:** Luisa Ronga, Iogann Tolbatov, Ester Giorgi, Paulina Pisarek, Christine Enjalbal, Alessandro Marrone, Diego Tesauro, Ryszard Lobinski, Tiziano Marzo, Damiano Cirri, Alessandro Pratesi

**Affiliations:** † Université de Pau et des Pays de l’Adour, E2S UPPA, CNRS, IPREM, 64053 Pau, France; ‡ Institute of Chemical Research of Catalonia (ICIQ), The Barcelona Institute of Science and Technology, Av. Paisos Catalans 16, 43007 Tarragona, Spain; § Department of Chemistry and Industrial Chemistry, University of Pisa, Via G. Moruzzi 13, 56124 Pisa, Italy; ∥ IBMM, Université de Montpellier, CNRS, ENSCM, UMR 5247, 34293 Montpellier, France; ⊥ Department of Pharmacy, University “G. D’Annunzio” Chieti-Pescara, Via dei Vestini, 31, 66100 Chieti, Italy; # Department of Pharmacy and CIRPeB, Università degli Studi di Napoli Federico II, 80131 Naples, Italy; ¶ Chair of Analytical Chemistry, Department of Chemistry, Warsaw University of Technology, Noakowskiego 3, 00-664 Warsaw, Poland; ∇ Department of Pharmacy, University of Pisa, Via Bonanno Pisano, 6, 56126 Pisa, Italy

## Abstract

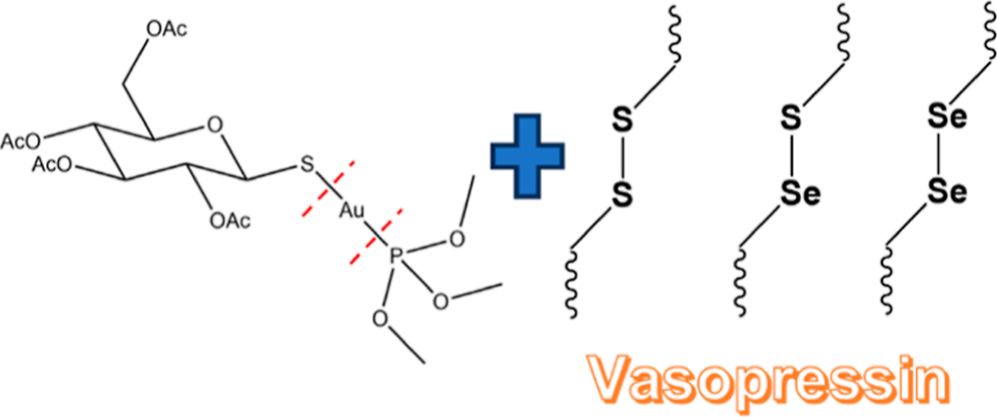

Auranofin, a gold(I)-based complex, is under clinical trials for application as an anticancer agent for the treatment of nonsmall-cell lung cancer and ovarian cancer. In the past years, different derivatives have been developed, modifying gold linear ligands in the search for new gold complexes endowed with a better pharmacological profile. Recently, a panel of four gold(I) complexes, inspired by the clinically established compound auranofin, was reported by our research group. As described, all compounds possess an [Au{P(OMe)_3_}]^+^ cationic moiety, in which the triethylphosphine of the parent compound auranofin was replaced with an oxygen-rich trimethylphosphite ligand. The gold(I) linear coordination geometry was complemented by Cl^–^, Br^–^, I^–^, and the auranofin-like thioglucose tetraacetate ligand. As previously reported, despite their close similarity to auranofin, the panel compounds exhibited some peculiar and distinctive features, such as lower log *P* values which can induce relevant differences in the overall pharmacokinetic profiles. To get better insight into the P–Au strength and stability, an extensive study was carried out for relevant biological models, including three different vasopressin peptide analogues and cysteine, using ^31^P NMR and LC-ESI-MS. A DFT computational study was also carried out for a better understanding of the theoretical fundamentals of the disclosed differences with regard to triethylphosphine parent compounds.

## Abstract 2

A combined experimental and theoretical approach has been used in order to assess how the structural modifications on the phosphine ligand of auranofin impact the chemical features of the resulting derivatives. These structural modifications were able to strongly affect the −Au−P− bond strength until obtaining an inverted order of ligand exchangeability with respect to the AF behavior, impacting the overall biological mode of action.

## Introduction

**Figure 1:**
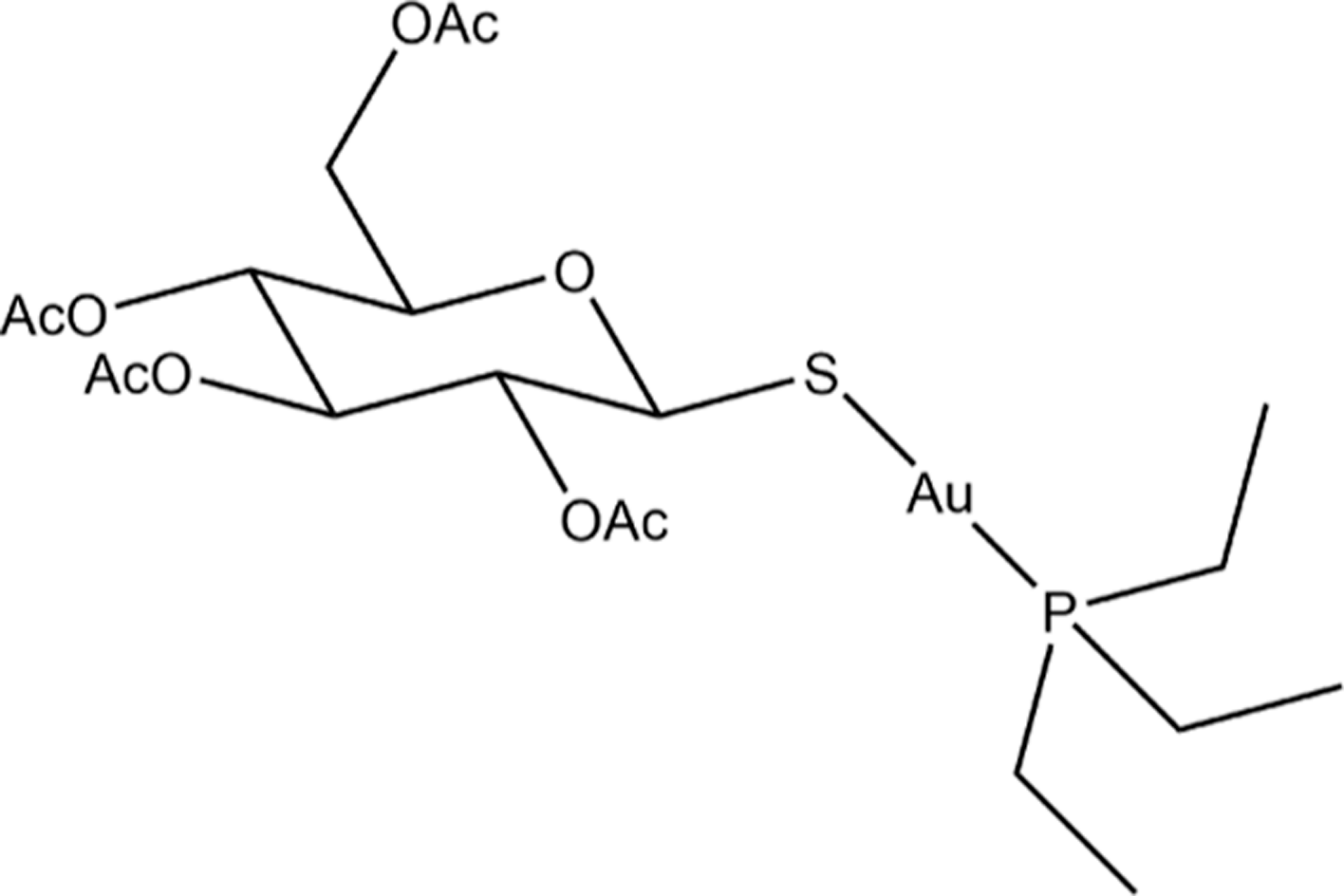
AF structure.

Recently, the so-called repurposing approach spurred a reappraisal of several approved drugs. In this context, various drugs’ repository screenings have been activated in search of new anticancer candidates. Some approved gold(I)-based drugs (e.g., sodium aurothiomalate, auranofin, and aurothioglucose) have been extensively investigated with promising results.^1^ Specifically, auranofin (AF hereafter), a gold(I)-based drug (Figure 1) approved by the FDA in 1985 for the treatment of severe forms of rheumatoid arthritis, is currently under clinical trials with the aim of repurposing for the treatment of nonsmall-cell lung cancer and ovarian cancer.^2^ Chemically, AF consists of a gold(I) center linearly coordinated with a triethylphosphine and a tetraacetylthioglucose ligands. AF behaves as a typical prodrug, requiring chemical activation before exerting biological actions. Indeed, the first step, involved in AF pharmacological activity, is the release of the thiosugar moiety. AF, once activated, can bind tightly to several biological targets, in particular, proteins containing free cysteines (Cys)^3−5^ or selenocysteines (Sec),^6,7^ producing cellular effects. AF, due to its antiproliferative properties in several tumoral models, became the reference compound for the emerging class of gold-based drugs.^8^

**Figure 2:**
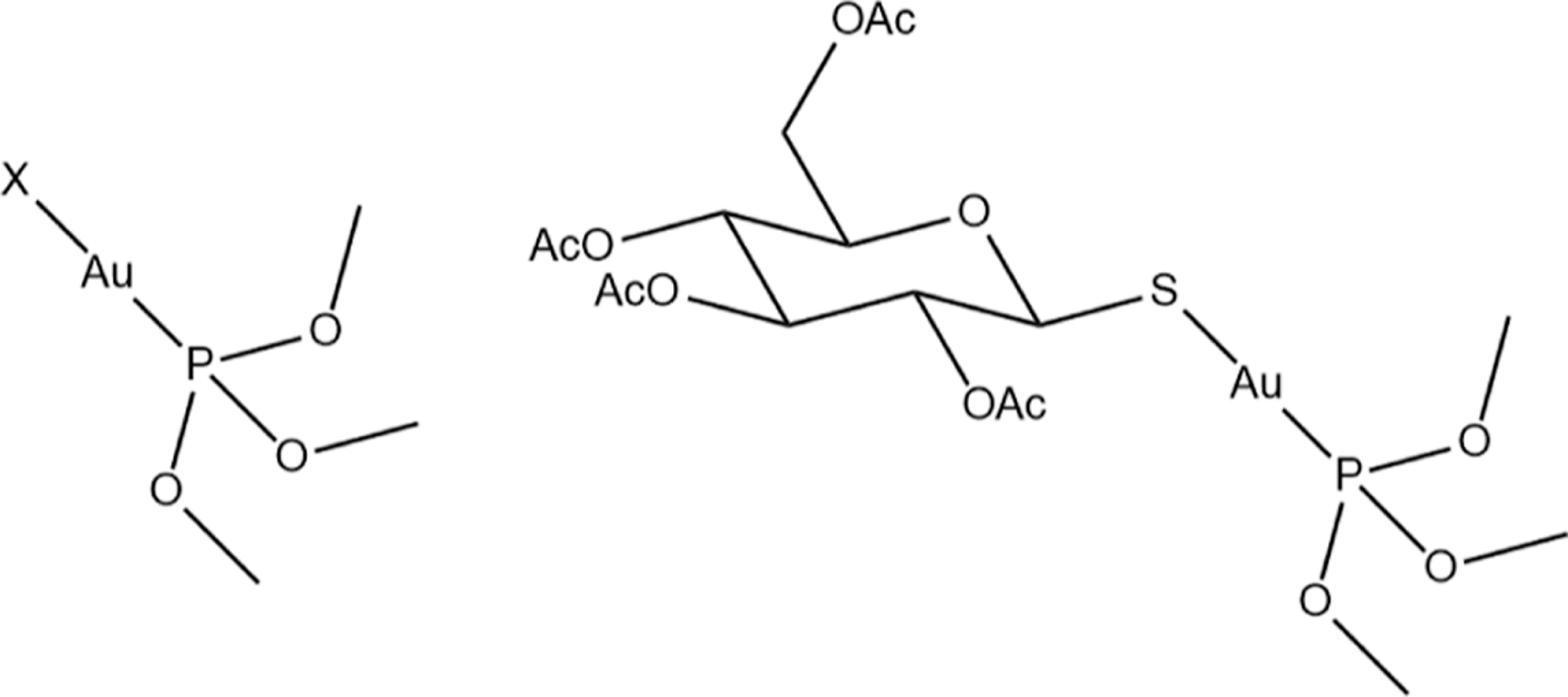
Investigated compounds. X = Cl, Br, I.

On the basis of these achievements, many efforts have been devoted to a systematic modification of its structure with the aim to obtain new derivatives with better pharmacological properties. Several modifications have been carried out, such as the preparation of halide derivatives, in which the thiosugar tetraacetate (STga) ligand was replaced with chloride, bromide, or iodide ions.^9^ An interesting modification was reported by Shaw, who replaced the sulfur of thiosugar with a selenium atom,^10^ while other investigations explored the effects of phosphine ligand modifications.^11^ We recently reported the synthesis of four novel AF derivatives, in which the triethylphosphine group was replaced with an oxygen-rich trimethylphosphite ester ligand (Figure 2).^12^

This panel of compounds showed some distinctive features, such as an improved water solubility compared to the triethylphosphine parent compounds and, most probably, a notable weakening of the gold–phosphorus bond. The latter hypothesis emerges from the previously published results, in which gold(I) trimethylphosphite derivatives were reported to interact with biological targets like human serum albumin and human carbonic anhydrase I usually through the formation of a simple protein–gold adduct—the term *adduct* denotes hereafter any species obtained by the coordination of a metal fragment with any other molecular fragment.^12^ Conversely, gold(I)-phosphine derivatives are known to interact through the formation of a protein-[Au(PR_3_)] adduct.^13^ To better elucidate this notable difference, we performed an extensive study by liquid chromatography (LC) coupled to electrospray high-resolution mass spectrometry (ESI-MS) in which gold(I)-trimethylphosphite derivatives were reacted toward representative peptide models bearing Cys and/or Sec residues in comparison with triethylphosphine parent complexes. LC-ESI-MS studies were integrated by a simple experimental procedure, in which the novel complexes were solubilized in the presence of the amino acid cysteine and the solution was monitored through ^31^P NMR spectroscopy. These two combined approaches highlighted relevant reactivity differences which are discussed below. Furthermore, quantum chemistry methodologies were employed for the comparative evaluation of the strength of the metal–ligand bonds between triethyl phosphine-containing AF and its trimethyl phosphite-based analogues. Indeed, DFT is a suitable tool for the characterization of the reaction pathways in the studies of the fate of metal ions and metallodrugs in the biological milieu.^14−17^

## Results

### LC–ESI-MS and ^31^P NMR Studies

**Figure 3:**
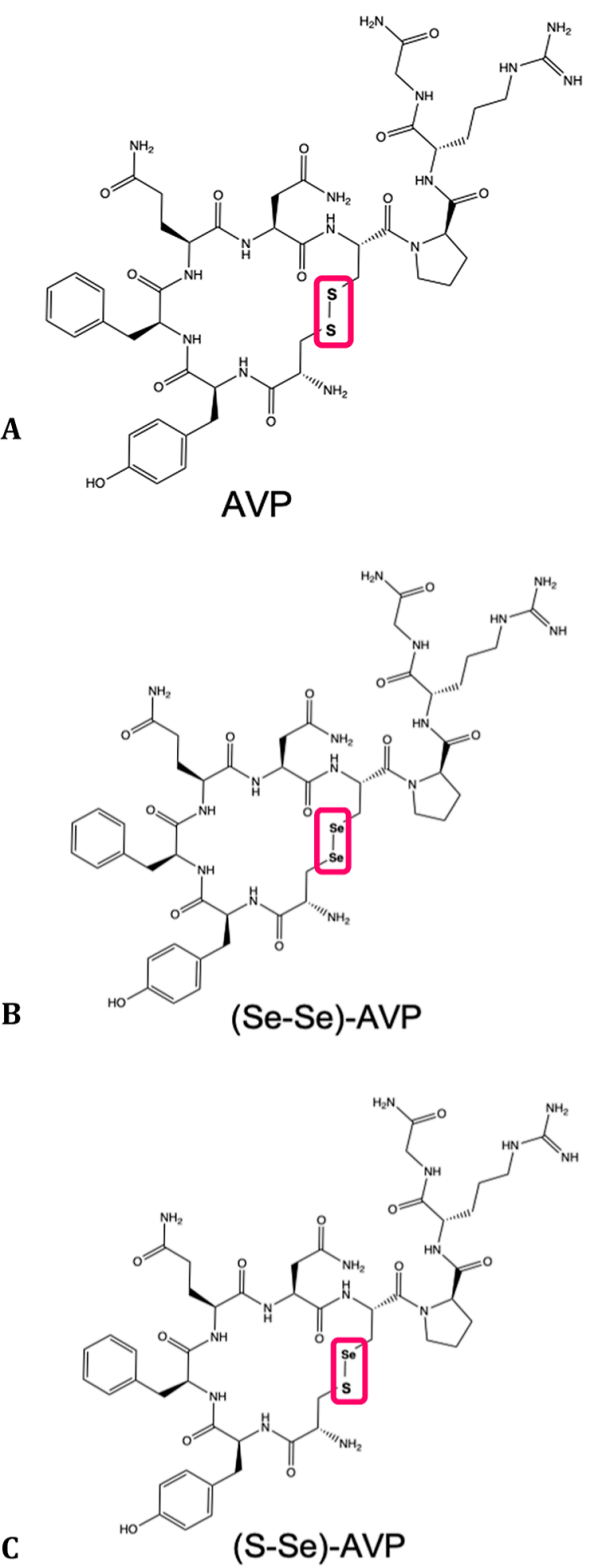
Vasopressin models employed for ESI–MS experiments.

The comparative reactivity of the gold phosphites with a model disulfide peptide (vasopressin, AVP), and its mono- and diselenium analogues (Figure 3) was investigated by LC–ESI–MS.

Vasopressin (Figure 3A) is a nonapeptide cyclized through two cysteine residues, best known for its antidiuretic and vasopressor actions.^18^ Its diselenide [(Se–Se)-AVP, Figure 3B] and selenylsulfide [(S–Se)-AVP, Figure 3C] analogues were previously described.^19−21^ These model peptides were proposed elsewhere for the study of the reactivity with medicinally and environmentally relevant metallic compounds.^21,22^

The three AVP model peptides were pretreated with the reducing agent dithiothreitol (DTT), to mimic the reducing cellular environment,^23^ and then reacted under physiological conditions (pH 7 and 37 °C) with AF and the gold phosphites [AuCl{P(OMe)_3_}], [AuBr{P(OMe)_3_}], [AuI{P(OMe)_3_}], and [Au{P(OMe)_3_}STga] at 1 to 3 peptide to Au molar ratio. The reaction of AVP with AF and its analogue [AuCl(PEt_3_)] was previously explored under the same conditions.^22^ The panel of reactions was therefore completed by incubating the (Se–Se)-AVP with [AuCl(PEt_3_)]. The reaction products were monitored by LC–ESI–MS and several peptide adducts were identified (Table S1 and Figures S1–S15). In Table 1, we report the main peptide adducts obtained by reacting each AVP peptide with this panel of Au(I) compounds. AF produced the +Au(I) peptide adduct by losing both its original ligands; only in the case of the (S–Se)-AVP, the +[Au(PEt_3_)]^+^ peptide adduct was observed. The latter adduct was also observed after the incubation of AVP and (Se–Se)-AVP with [AuCl(PEt_3_)]. The possible formation of biomolecules/Au(I) adducts with the complete loss of the ligands is a quite common behavior for gold(I)/(III)-based compounds.^24,25^

**Table 1:** Peptide Adduct Detected by LC-ESI-MS by Reacting AVP Peptides with AF and Its Analogues^a^

	triethylphosphine		trimethylphosphite	
	STga	Cl	STga	X^d^
AVP	+Au^b^	+Au; +[Au(PEt_3_)]^c^	+Au(I) (traces)	+Au
(Se–Se)-AVP	+Au	+Au; +[Au(PEt_3_)]	+ 2 O=P(OMe)_2_	+Au
(S–Se)-AVP	+Au; +[Au(PEt_3_)]	not performed	+ O=P(OMe)_2_ + STga	+Au

a Semicolons separate different adducts.

b Figure S13 of ref (22).

c Figure S14 of ref (22).

d Referred to the chloride-, bromide-, or iodide-containing analogues.

Concerning the reactivity of gold phosphites, the three [AuX{P(OMe)_3_}] compounds exclusively generated the +Au(I) peptide adducts, while the [Au{P(OMe)_3_}STga] showed a peculiar reactivity: only traces of the +Au(I) adduct were formed in the reaction with AVP, while no metalation was detected in the case of (Se–Se)- and (S–Se)-AVP. After incubation of [Au{P(OMe)_3_}STga] with these two AVP selenium-containing analogues, the formation of adducts between the peptides and the gold ligands was observed. More in detail, we observed an almost quantitative conversion of the (Se–Se)-AVP into an adduct with two dimethyl phosphite moieties (+O=P(OMe)_2_) bound to the two selenium atoms. Moreover, the (S–Se)-AVP was almost totally converted into an adduct with a dimethyl phosphite and a thiosugar (+STga) linked to the selenium and the sulfur, respectively.

**Figure 4:**
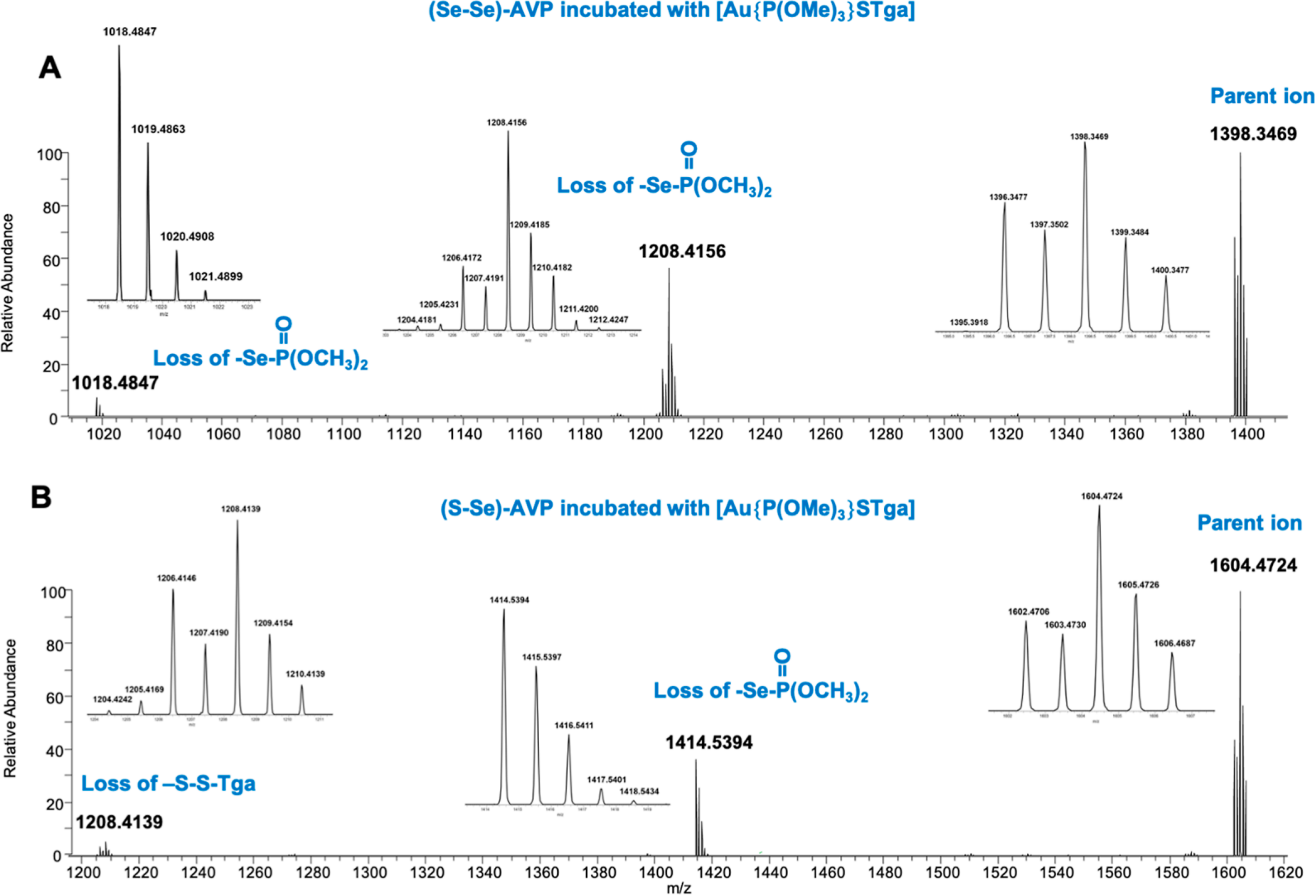
(Se–Se)-AVP (A) and (S–Se)-AVP (B) incubated with 3 equiv of [Au{P(OMe)_3_}STga] at 37 °C in the presence of DTT for 18 h. (A) MS/MS at *m/z* 1398.3414 (*z* = 1, isolation width = 5.0 *m/z*.): principal fragments at HCD = 20. (B) MS/MS at *m/z* 1604.4667 (*z* = 1, isolation width = 5.0 *m/z*): principal fragments at HCD = 20.

As it is shown in Figure 4, the binding sites between the ligands and the peptides were elucidated by the fragmentation of these adducts in the gas phase (MS^2^).

**Figure 5:**
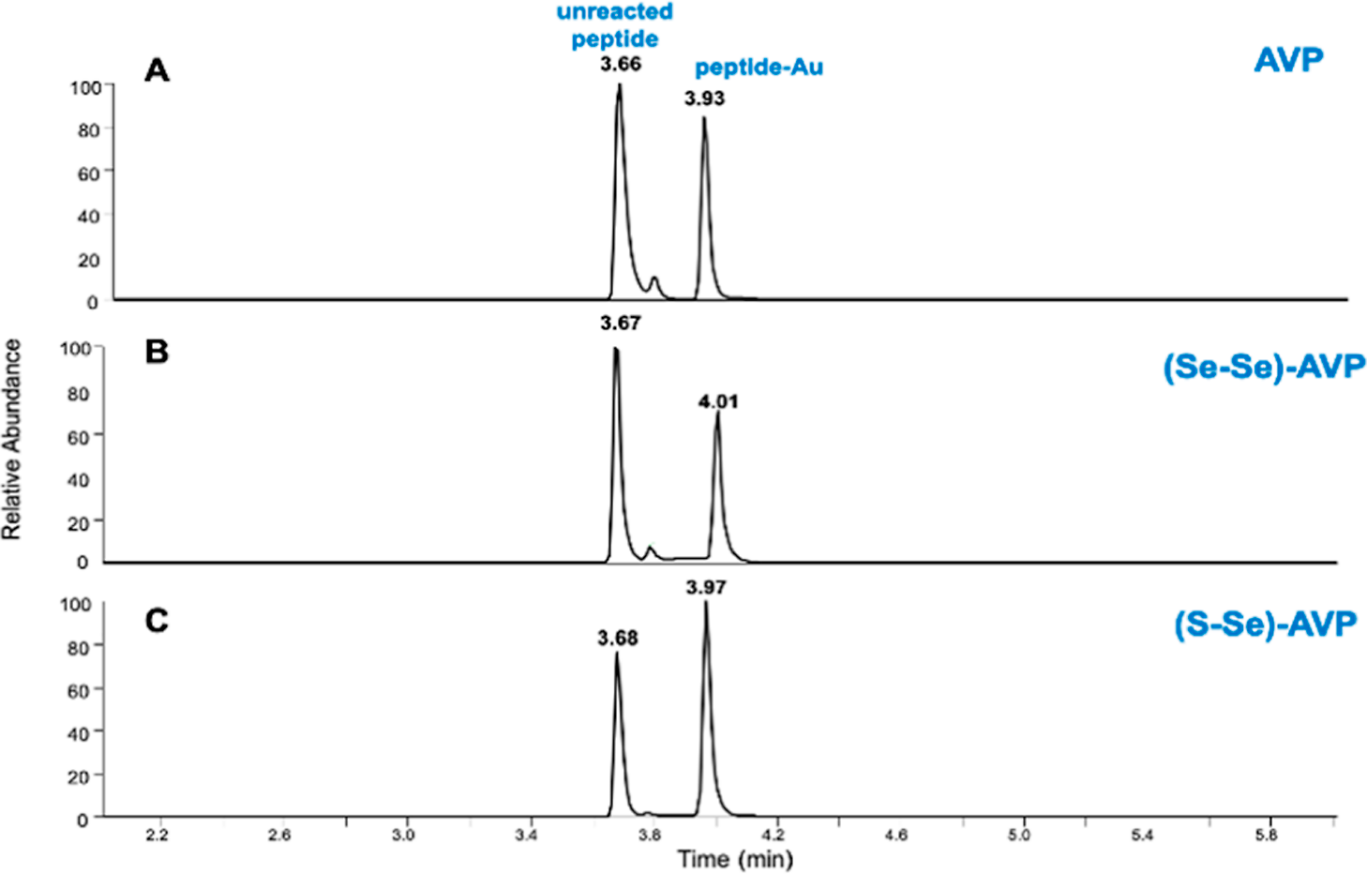
LC–MS of AVP peptides incubated with 3 equiv of [AuCl{P(OMe)_3_}] at 37 °C in the presence of DTT during 18 h: (A) AVP: XIC of ions *m/z* 543.72 (*z* = 2, unreacted peptide, *t*_R_ = 3.66) and 641,73 (*z* = 2, peptide-Au adduct, *t*_R_ = 3,93); (B) (Se–Se)-AVP: XIC of ions *m/z* 590.67 (*z* = 2, unreacted peptide, *t*_R_ = 3.68) and 689.65 (*z* = 2, peptide-Au adduct, *t*_R_ = 4.00); (C) (S–Se)-AVP: XIC of ions *m/z* 566,70 (*z* = 2, unreacted peptide, *t*_R_ = 3.68) and 665,67 (*z* = 2, peptide-Au adduct, *t*_R_ = 3,97).

In the case of diselenide AVP (Figure 4A), the progressive fragmentation of two −Se–P(O)–(OMe)_2_ moieties from the parent ion with the consequent loss of two consecutive Se isotopic patterns was observed. In the case of the selenylsulfide AVP (Figure 4B), the fragmentations of −Se–P(O)–(OMe)_2_ (with the loss of Se isotopic pattern) and −S–STga moieties from the parent ion were detected. This comparative study of the reactivity of cysteinyl and selenocysteinyl peptides with trimethylphosphite and triethylphosphine gold(I) compounds allowed us to demonstrate that, within peptide metalation, the gold was able to retain the phosphorus ligand only in the case of triethylphosphine-containing compounds. In contrast to that, in the case of phosphite compounds, the trimethylphosphite ligand was lost and an adduct with the bare gold(I) ion was produced. This is likely to be related to the higher labile character of the P(OMe)_3_ ligand than that of PEt_3_. Among all the tested gold(I) compounds, [Au{P(OMe)_3_}STga] revealed a distinct feature: it was not able to metalate the peptides (traces of the +Au adduct were observed only in the case of AVP), but it transferred the phosphite ligand to the Se of Sec and the thiosugar to the S of Cys. This analysis also demonstrated a similar reactivity of [AuCl{P(OMe)_3_}], [AuBr{P(OMe)_3_}], and [AuI{P(OMe)_3_}], leading to an identical extent of metalation of the same AVP peptide (Figures S2–S4, S8–S10, and S13–S15). Moreover, it appeared that these [AuX{P(OMe)_3_}] compounds gave the highest metalation on the (S–Se)-AVP peptide. Indeed, in Figure 5 are compared the LC–MS spectra of the three AVP peptides reacted with [AuCl{P(OMe)_3_}], chosen as an example.

In order to understand this different behavior toward peptide targets, we decided to perform some ^31^P NMR experiments. For this reason, all panel compounds were allowed to interact with Cys, and the samples were acquired at t_0_ and after 24 h of incubation at 37 °C.

The panel compounds showed very different reaction patterns in comparison with triethylphosphine derivatives. All of them turned out not to form Cys-S-[Au(PR_3_)] adducts, which is an extensively reported behavior in the case of AF-like complexes,^13,26,27^ and was confirmed also in this study (see the Supporting Information, Figures S16–S23). Conversely, all the investigated complexes underwent an activation that relies on the trimethylphosphite ligand release. This latter observation was confirmed owing to the presence of multiple signals, some of them attributable to trimethylphosphite in the same solvent (four peaks between 14 and 16 ppm), while others related to side-reaction byproducts (see the Supporting Information, Figures S24–S32).

## DFT Calculations

The results of the ESI-MS and ^31^P NMR experiments suggested a general weakening of the gold–phosphorus coordinative bond, which could be qualitatively explained through the higher inductive effect, with respect to AF, due to the introduction of oxygen atoms. For this reason, the strength of the gold–phosphorus bond and thus the impact of the ligand substitution in the complexes of AF and [Au{P(OMe)_3_}STga] were assessed by means of DFT calculation. The strength of Au–P and Au–S bonds was assessed through the calculation of the corresponding snapping energies, i.e., energies for the dissociation into unrelaxed fragments, bond dissociation enthalpies (BDEs), and bond dissociation free energies (BDFEs) (Table 2). While snapping energies and BDEs provide for quantification of the bond strength, the BDFE values, due to the inclusion of the −TΔS term, also estimate the thermodynamic feasibility of the Au–P and Au–S dissociation. It was determined that the substitution of the triethylphosphine ligand and trimethylphosphite altered the reactivity mechanism of the metallodrug completely. In AF, the phosphine-based ligand plays the role of the carrier ligand, whereas the thioglucose is the labile ligand easily cleaved. These experimental observations were well corroborated by the data presented in Table 2, i.e., the snapping energy and BDE values for the breaking of the Au–S bond in auranofin were substantially lower than those for Au–P, by 5–7 kcal/mol. Moreover, the entropy-related BDFE values yielded a slightly smaller difference, describing the Au–S bonds as more labile by ∼5 kcal/mol. On the other hand, the AF analogue based on P(OMe)_3_ showed a different behavior: the Au–P bond was greatly weakened by the presence of oxygens. This could be explained by the enhanced electron attraction exerted by the three oxygen atoms that lowered the electron density of the phosphorus atom. Thus, we consider that the P lone pair involved in the σ-donation to the metal center is less effective in the P(OMe)_3_ derivatives compared to AF. Indeed, the snapping energies for the Au–P and Au–S bonds were found to be 53.0 *vs.* 56.3 kcal/mol. Similar differences of 5–6 kcal/mol were observed for BDE and BDFE values.

**Table 2:** Snapping Energies, BDEs, and BDFEs^a^

complex	bond	snapping energy	BDE	BDFE
Auranofin	Au–P	59.1	54.7	43.7
	Au–S	52.6	49.8	39.0
[Au{P(OMe)_3_}STga]	Au–P	53.0	47.4	36.7
	Au–S	56.3	53.2	42.2

a All values are reported in kcal/mol.

Moreover, the analysis of Mulliken charge distribution in AF and [Au{P(OMe)_3_}STga] was carried out to look deeper into the source of the bond strength alteration (Table 3). The substitution of the triethylphosphine ligand with the trimethylphosphite one affected the charge distribution on the whole complex. More precisely, the phosphorus ligand was found to bear a lower charge in the trimethylphosphite derivative, +0.30, compared to the native AF, +0.37. The phosphorus atom charge increased drastically from +0.17 to +0.60 passing from the native to the phosphite derivative of AF as an effect of the electron attraction exerted by the P-bound oxygen atoms. The charge on gold increased from +0.14 to +0.20. The charge on sulfur changed marginally, decreasing from −0.51 to −0.50, whereas the charge on the whole thioglucose fragment altered marginally as well, going from −0.51 to −0.60. These data indicated that the diminished strength of the Au–P bond in [Au{P(OMe)_3_}STga] could be also explained by the less favorable electrostatics compared to the same bond in AF. The concomitance of the decreased availability of the P lone pair and less favorable electrostatics explain the weakening of the Au–P bond in the phosphite derivative and the consequent carrier/labile roles’ inversion of P(OMe)_3_ with respect to the PEt_3_ ligand.

**Table 3:** Mulliken Charge Distribution in Auranofin and [Au{P(OMe)_3_}STga]^a^

	Mulliken charges	
fragment or atom	Auranofin	[Au{P(OMe)_3_}STga]
Au	+0.14	+0.20
P	+0.17	+0.60
P-containing ligand PEt_3_ or P(OMe)_3_	+0.37	+0.30
S	–0.51	–0.50
Thioglucose	–0.51	–0.60

a All values are reported in a.u.

Thus, the comparison of bond enthalpies and free energies in two complexes corroborated well the experimental evidence, showing that the appreciable lowering of the Au–P strength in the AF analogue [Au{P(OMe)_3_}STga] and the subsequent acquisition of the labile character of the P(OMe)_3_ ligand were the reasons behind the difference in the biomolecular activity of [Au{P(OMe)_3_}STga].

This accurate evaluation of bond strengths showed crucial differences in the metal–ligand interaction between two complexes bearing the thiosugar tetraacetate anionic ligand (STga in raw formulas hereafter). Indeed, the thiosugar of AF is a labile ligand, according to both our calculations and the general consensus in the field,^26,28^ while its triethylphosphine represents a stable ligand; i.e., it remains coordinated to Au(I) after the cleavage of thioglucose in the [Au(PEt_3_)]^+^ cation, which is considered to be the active species originating from AF. On the other hand, the substitution of triethylphosphine with trimethylphosphite strengthens the Au–S bond by making the phosphite ligand more promptly exchangeable than the thiosugar. This drastic change in the strength of metal–ligand bonds is expected to significantly impact the reactivity of Au(I)–phosphite complexes with biomolecular targets, thus leading to different biological responses compared to AF.

## Experimental Section

### Complex Preparation and Characterization

The investigated compounds came from the same batches used in our recently published work and were therefore already prepared and characterized.^12^

### LC–MS Experiments

#### Sample Preparation

Stock solutions of AVP, (Se–Se)-AVP, (S–Se)-AVP 0.9 mM, and DTT 0.2 M were prepared by dissolving the samples in ultrapure water. An ammonium acetate buffer solution (2 mM, pH 7.0) was prepared by weighing ammonium acetate and dissolving it in ultrapure water, pH adjustment was carried out with acetic acid and ammoniac commercial solutions. For the incubation with gold(I) compounds, stock solutions at 10 mM AF, [AuCl(PEt_3_)], [AuCl{P(OMe)_3_}], [AuBr{P(OMe)_3_}], [AuI{P(OMe)_3_}], and [Au{P(OMe)_3_}STga] were prepared by dissolving the samples in DMSO. For the prereduction of peptides, aliquots of their stock solution were diluted with a 2 mM ammonium acetate solution (pH 7.0) to a 0.1 mM final peptide concentration. Then, aliquots of DTT stock solution were added in peptide to reducing agent ratios of 1:10 (final concentration of 1 mM of the reducing agent) and the mixtures were incubated for 30 min at 37 °C in a water bath under stirring. Later, aliquots of AF, [AuCl(PEt_3_)]. [AuCl{P(OMe)_3_}], [AuBr{P(OMe)_3_}], [AuI{P(OMe)_3_}], and [Au{P(OMe)_3_}STga] stock solutions were added in peptide to Au ratios of 1:3 [0.3 mM Au(I) concentration]. The mixtures were left under stirring overnight at 37 °C in a water bath. After the incubation, all the Au(I) incubated solutions were sampled and diluted to a final peptide concentration of 6 μM using 2 mM ammonium acetate with pH 7.0 and 2% (v/v) of acetonitrile (AcN) and used for LC–MS analysis.

#### LC–ESI–MS

LC separations were performed using Dionex Ultimate 3000 series UHPLC (Thermo Fisher Scientific) coupled to an Orbitrap Q-Exactive Plus Mass Spectrometer (Thermo Fisher Scientific). The used column was Acclaim 120 (C18, 5 μm, 120 Å, 4.6 mm × 100 mm) (Thermo Fisher Scientific). The mobile phases were A, H_2_O, and B, AcN, both with 0.1% formic acid. The flow rate used in all LC–MS experiments was 1 mL·min^–1^, and sample elution was performed by using the gradient from 5 to 95% of B over 6.5 min. 10 μL of sample injection was set. Ionization was performed using an electrospray ion source operating in positive ion mode with a capillary voltage of 3.80 kV and a capillary temperature of 400 °C. The sheath gas, auxiliary gas, and sweep gas flow rates were set at 75, 20, and 1 (arbitrary units), respectively. The auxiliary gas temperature was set at 500 °C. For each studied mass, a set of MS/MS spectra were acquired using an isolation width of 1.0 and 5.0 *m/z*, and a screening of collision energies (from 10 to 40 HCD) was carried out. All MS^n^ data were analyzed using the Qual Browser embedded in the Thermo Fisher Scientific Xcalibur program and the FreeStyle software.

### ^31^P NMR Experiments

All NMR spectra were acquired on a JEOL400YH spectrometer (resonating frequencies for ^31^P 160 MHz) at room temperature (25 ± 2 °C) in D_2_O with a deuteration degree of 99.9%. Samples for testing cysteine interaction were prepared as follows: 1.57 mg (0.00890 mmol) of the Cys·HCl monohydrate were moved in an Eppendorf test tube and solubilized with 430 μL of D_2_O; subsequently, 70 μL of a 0.29 M DMSO metal complex solution was added. Final concentrations were 1.79 × 10^–2^ M for Cys and 2.00 × 10^–2^ M for metal complexes. Each sample was moved to a standard NMR tube and acquired with a classical power-gated 90 deg pulse sequence (128 scans; 2 s recycle delay). A sample for ^31^P NMR control spectrum of trimethyl phosphite (Figure S32) was prepared as follows: one drop of trimethylphosphite was added to an Eppendorf tube and mixed with 70 μL of DMSO. The resulting mixture was diluted with 430 μL of D_2_O and then moved to an NMR tube for spectrum acquisition.

### DFT Calculations

All DFT computations were performed in the Gaussian 16 C.01 quantum chemistry package.^29^ DFT is a ubiquitous tool for the characterization of structures of metal complexes.^30−33^ The def2TZVP basis set^34^ and the water solvation model (IEFPCM)^35^ were employed in all the geometrical optimizations, single-point electronic, and solvation energy calculations. The range-corrected ωB97X hybrid density functional was utilized^36^ because it yields geometries and reaction profiles for transition-metal-containing compounds with improved accuracy.^37,38^ The frequency computations substantiated the true character of the stationary points and permitted the assessment of zero-point energy and thermal corrections. The employed IEFPCM continuum solvent method produces significantly smaller errors than other continuum models for aqueous free energies of solvation for cations, anions, and neutrals, being especially robust for the computation of solution properties requiring precise assessment of solution free energies.^39^ The strength of Au–P and Au–S bonds in the studied complexes was characterized via the computation of the corresponding snapping energies, i.e., the energies for the dissociation into unrelaxed fragments, BDEs, and BDFEs. Snapping energies were obtained by subtracting the single-point energies of the two bonding fragments at the geometry taken by the complex from the electronic energy of the whole optimized complex. BDEs/BDFEs are the differences between the enthalpy/free energy of the fully optimized complexes and the enthalpies/free energies of the bonding fragments after relaxation.

## Conclusions

We have exploited a combined experimental and theoretical approach to assess how the structural modification on the phosphine ligand of AF and some of its established analogues—i.e., the insertion of oxygen atoms—impacts the chemical features of the resulting derivatives. DFT calculations unveiled the inverted order of ligand exchangeability in the [Au{P(OMe)_3_}STga] complex compared to AF that is expected to impact the biological mode of action of this Au(I) compound, thus corroborating the experimental evidence. It was found that a complete reversal of the metal–ligand bond strengths occurs: while thioglucose and triethylphosphine are labile and carrier ligands in the AF complex, respectively, the thioglucose and trimethylphosphite ligands become instead the carrier and the labile ligands in the [Au{P(OMe)_3_}STga] complex. We would like to stress that the thiosugar moiety in AF is not relevant for the cytotoxicity effects; however, it also contributes to imparting some specific properties to the complex, including the capability to form noncovalent interaction with some proteins. Conversely, AF analogues in which the thiosugar moiety is replaced with different ligands (e.g., halides) do not give this kind of interaction.^40^ Accordingly, the release of the phosphite—instead of the thiosugar—may modulate the pharmacology of the obtained molecule. Altogether, the inversion in the carrier/labile role of the Au-bound ligands in the phosphite analogue of AF suggests that the modulation of the inductive features of the Au-bound ligands may be employed to control the ligand exchange processes that may occur in the biological milieu.

## Supporting Information Available

Chromatograms and mass spectra and ^31^P NMR spectra (PDF)
