# Supplementary material for: Mechanistic Evaluations of the Effects of Auranofin Triethylphosphine Replacement with a Trimethylphosphite Moiety
Source: Inorg Chem. 2023 Jun 21;62(26):10389–96. doi: 10.1021/acs.inorgchem.3c01280 (PMC10324304; doi:10.1021/acs.inorgchem.3c01280)
Supplement: Supplementary file 1 — ic3c01280_si_001.pdf [file ic3c01280_si_001.pdf]

Supporting Information

Mechanistic evaluations of the effects of auranofin triethylphosphine replacement with a trimethylphosphite moiety

Luisa Ronga[1,#], Iogann Tolbatov[2,#], Ester Giorgi[3], Paulina Pisarek[1], Christine Enjalbal[4], Alessandro Marrone[5], Diego Tesauro[6], Ryszard Lobinski[1,7], Tiziano Marzo[8], Damiano Cirri[3,*], Alessandro Pratesi[3,*]

1 Université de Pau et des Pays de l'Adour, E2S UPPA, CNRS, IPREM, 64053 Pau, France
2 Institute of Chemical Research of Catalonia (ICIQ), The Barcelona Institute of Science and Technology, Av. Paisos Catalans 16, 43007 Tarragona, Spain
3 Department of Chemistry and Industrial Chemistry, University of Pisa, Via G. Moruzzi 13, 56124 Pisa, Italy
4 IBMM, Université de Montpellier, CNRS, ENSCM, UMR 5247, 34293 Montpellier, France
5 Department of Pharmacy, University "G. D'Annunzio" Chieti-Pescara, Via dei Vestini, 31, 66100 Chieti, Italy
6 Department of Pharmacy and CIRPeB, Università degli Studi di Napoli Federico II, 80131 Naples, Italy
7 Chair of Analytical Chemistry, Department of Chemistry, Warsaw University of Technology, Noakowskiego 3, 00-664 Warsaw, Poland.
8 Department of Pharmacy, University of Pisa, Via Bonanno Pisano, 6, 56126 Pisa, Italy

# These authors equally contributed.

Corresponding Authors: Damiano Cirri, damiano.cirri@unipi.it; Alessandro Pratesi, alessandro.pratesi@unipi.it

Index:

LC-ESI-MS data S2
Triethylphosphine complexes ^{31}P NMR data S11
Trimethylphosphite complexes ^{31}P NMR data S15
Trimethylphosphite ^{31}P NMR spectrum S19

LC-ESI-MS data

Table S1. List of observed peptide adducts by MS.

Peptide	Formula	Theoretical monoisotopic mass (Da)	Experimental mass of the most abundant isotope ($Se^{79.9160}$) (Da)	Observed most abundant ions *m/z*	
				z=1	*z*=2
AVP	$C_{46}H_{65}N_{15}O_{12}S_2$	1083.4379	1083.4368	1084.4441	542.7257
Reduced AVP	$C_{46}H_{67}N_{15}O_{12}S_2$	1085.4535	1085.4525	1086.4598	543.7340
AVP + Au	$C_{46}H_{66}HAuN_{15}O_{12}S_2$	1281.4122	1281.4157	1282.4157	641.7123
(Se-Se)-AVP	$C_{46}H_{65}N_{15}O_{12}Se_2$	1179.3266	1179.3266	1180.3339	590.6707
(Se-Se)-AVP + Au	$C_{46}H_{66}AuN_{15}O_{12}Se_2$	1377.3011	1377.3069	1378.3069	689.6563
(Se-Se)-AVP+AuPEt$_3$	$C_{52}H_{78}AuN_{14}O_{13}PSe$[a]	1414.4679	1414.4486	1415.4486	708.2243
(Se-Se)-AVP + 2P(O)(OCH$_3$)$_2$	$C_{50}H_{77}N_{15}O_{18}P_2Se_2$	1397.3377	1397.3412	1398.3412	699.6743
(S-Se)-AVP	$C_{46}H_{65}N_{15}O_{12}SSe$	1131.3823	1131.3804	1132.3877	566.6975
(S-Se)-AVP + Au	$C_{46}H_{66}AuN_{15}O_{12}SSe$	1329.3567	1329.3686	1330.3599	665.6843
(S-Se)-AVP + AuPEt$_3$	$C_{52}H_{81}AuN_{15}O_{12}SSe$	1447,4478	1447.4522	1448,4522	724,7304
(S-Se)-AVP + P(O)(OCH$_3$)$_2$+STga	C62H90N15O24PS$_2$Se	1603.4627	1603.4661	1604.4661	802.7374

[a] formation at the N-terminus of a pyruvoyl group, a hydrolysis product of the unsaturated amino acid dehydroalanine, formed by β-elimination of Sec.[16]

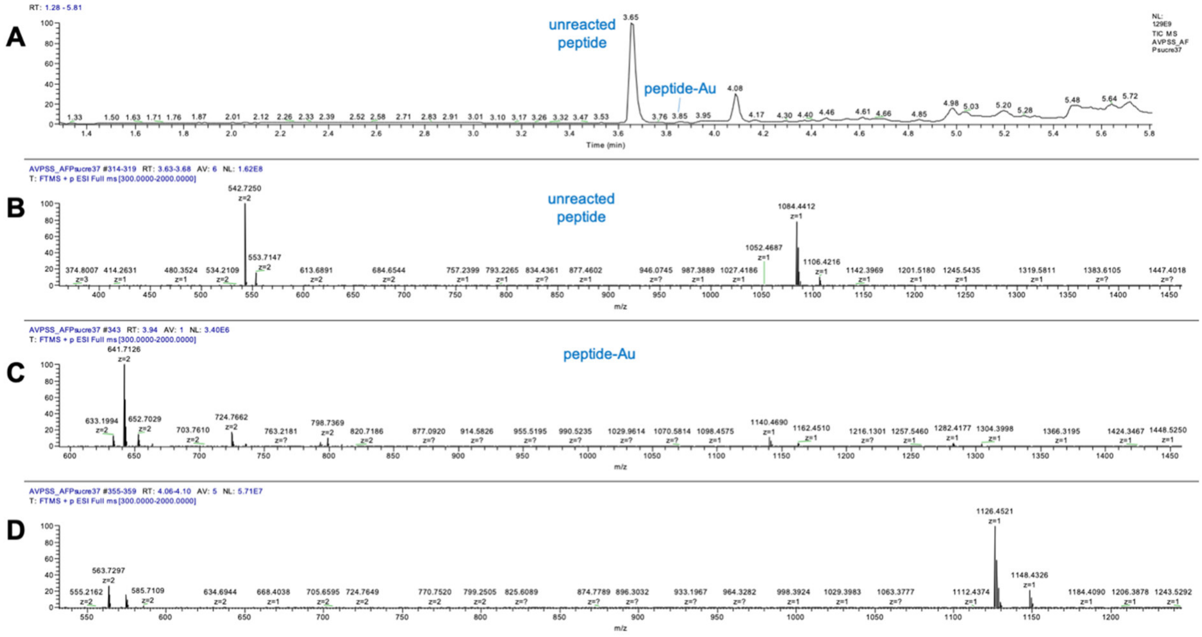

Figure S1. LC-MS of AVP incubated 18 h with [Au{P(OMe)$_3$}STga] (3 eq.) at 37 °C in presence of DTT. (**A**) TIC. (**B**) Mass spectrum of peak at t_R = 3.65 min. (**C**) Mass spectrum of peak at t_R = 3.95 min. (**D**) Mass spectrum of peak at t_R = 4.08 min.

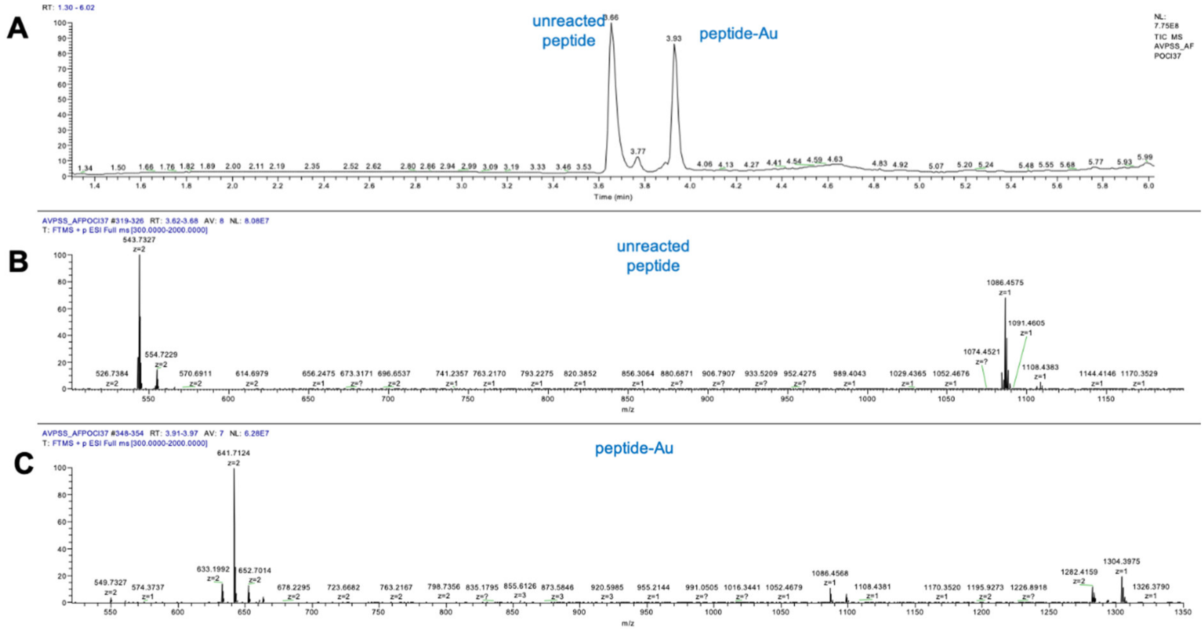

Figure S2. LC-MS of AVP incubated 18 h with [AuCl{P(OMe)$_3$}] (3 eq.) at 37 °C in presence of DTT. (**A**) TIC. (**B**) Mass spectrum of peak at t_R = 3.65 min. (**C**) Mass spectrum of peak at t_R = 3.92 min.

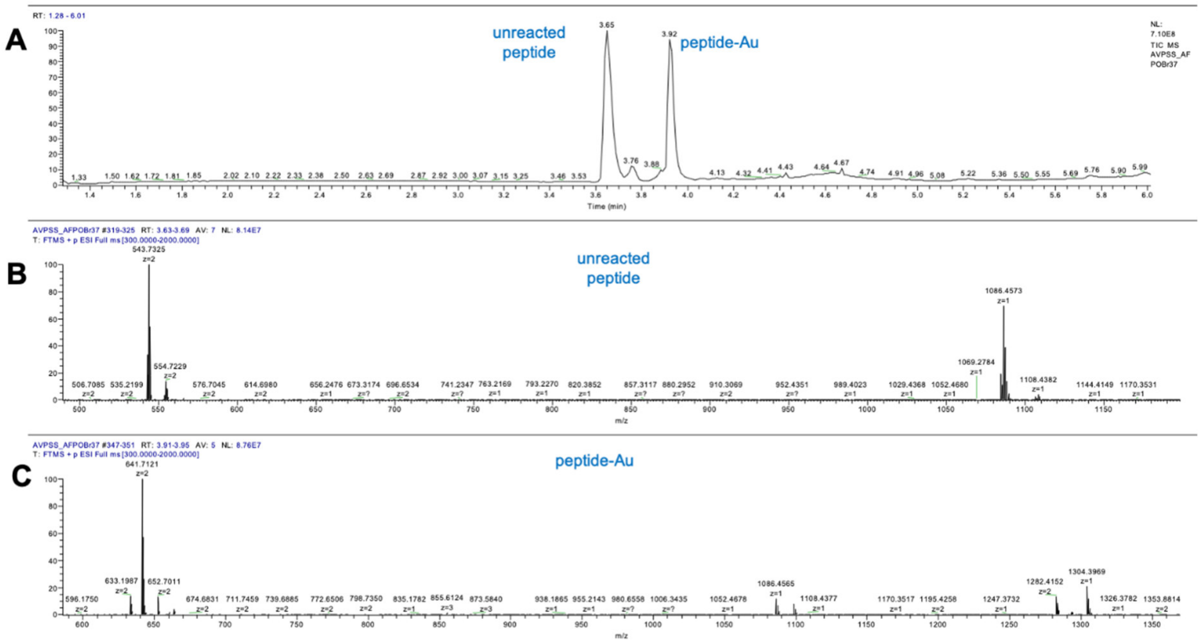

Figure S3. LC-MS of AVP incubated 18 h with [AuBr{P(OMe)$_3$}] (3 eq.) at 37 °C in presence of DTT. (**A**) TIC. (**B**) Mass spectrum of peak at t_R = 3.65 min. (**C**) Mass spectrum of peak at t_R = 3.92 min.

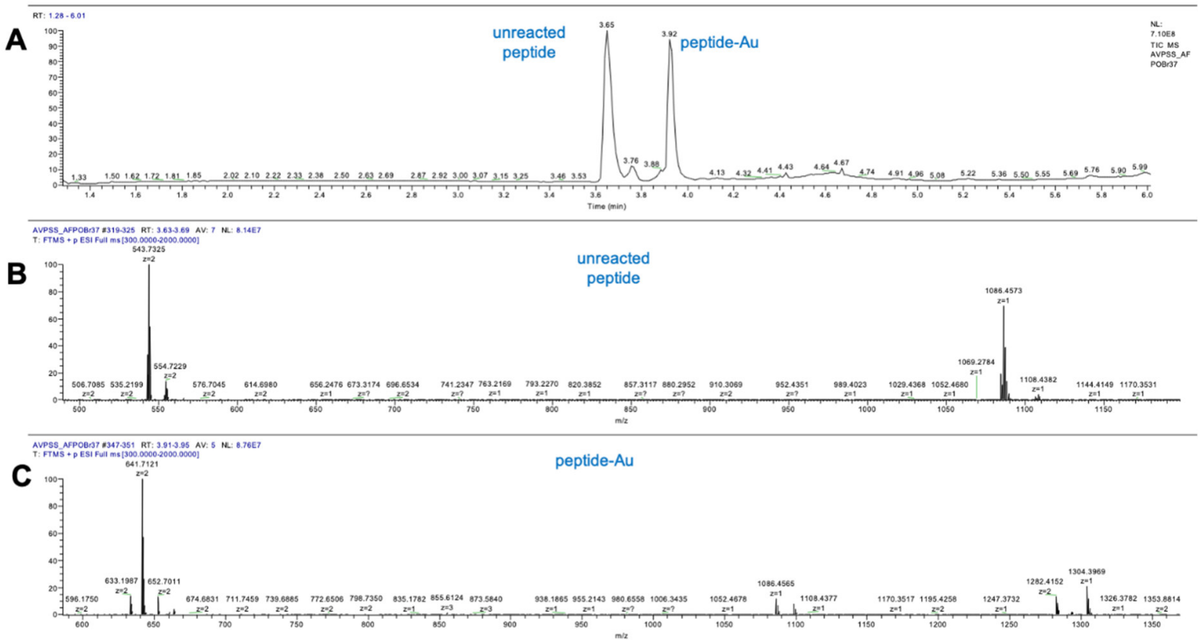

Figure S4. LC-MS of AVP incubated 18 h with [AuI{P(OMe)$_3$}] (3 eq.) at 37 °C in presence of DTT. (**A**) TIC. (**B**) Mass spectrum of peak at t_R = 3.65 min. (**C**) Mass spectrum of peak at t_R = 3.92 min.

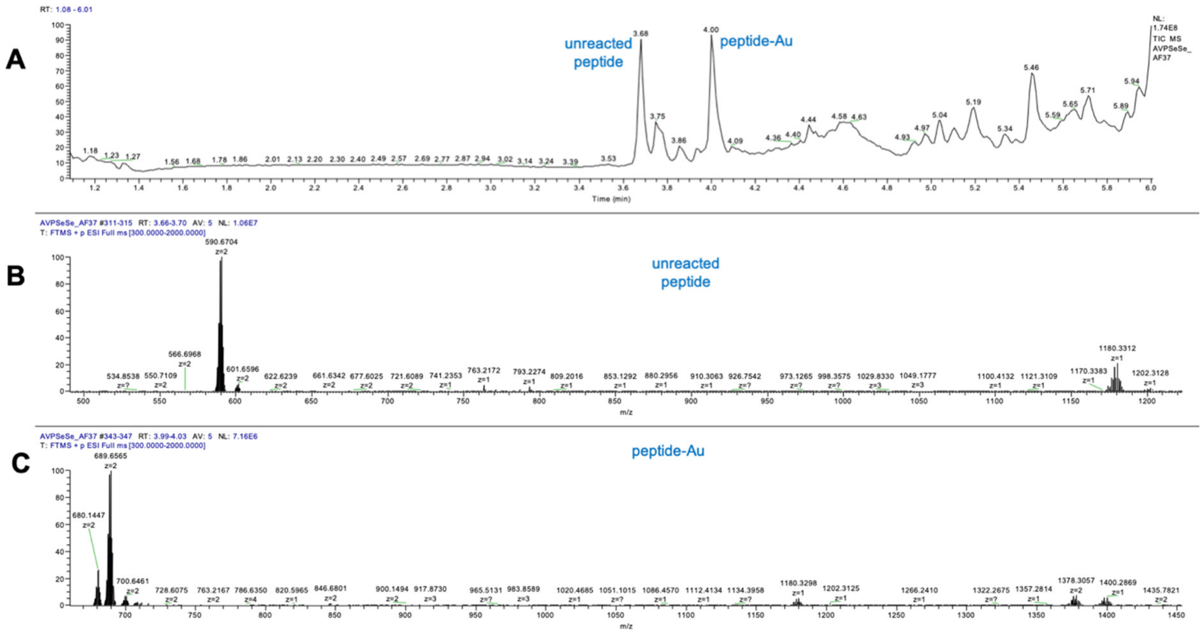

Figure S5. LC-MS of (Se-Se)-AVP incubated 18 h with AF (3 eq.) at 37 °C in presence of DTT. (**A**) TIC. (**B**) Mass spectrum of peak at t_R = 3.68 min. (**C**) Mass spectrum of peak at t_R = 4.00 min.

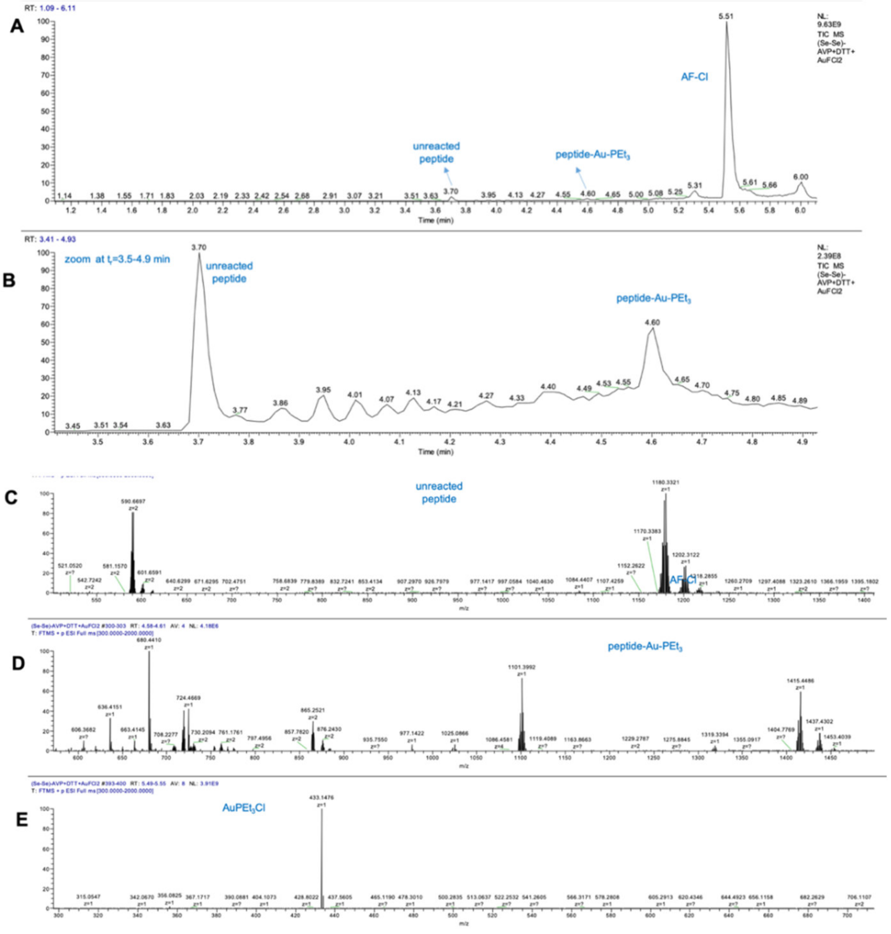

Figure S6. LC-MS of (Se-Se)-AVP incubated 18 h with [AuCl(PEt_3)] (3 eq.) at 37 °C in presence of DTT. (**A**) TIC. (**B**) Zoom of TIC at t_R = 3.5-4.9 min. (**C**) Mass spectrum of peak at t_R = 3.70 min. (**D**) Mass spectrum of peak at t_R = 4.60 min. (**D**) Mass spectrum of peak at t_R = 5.50 min.

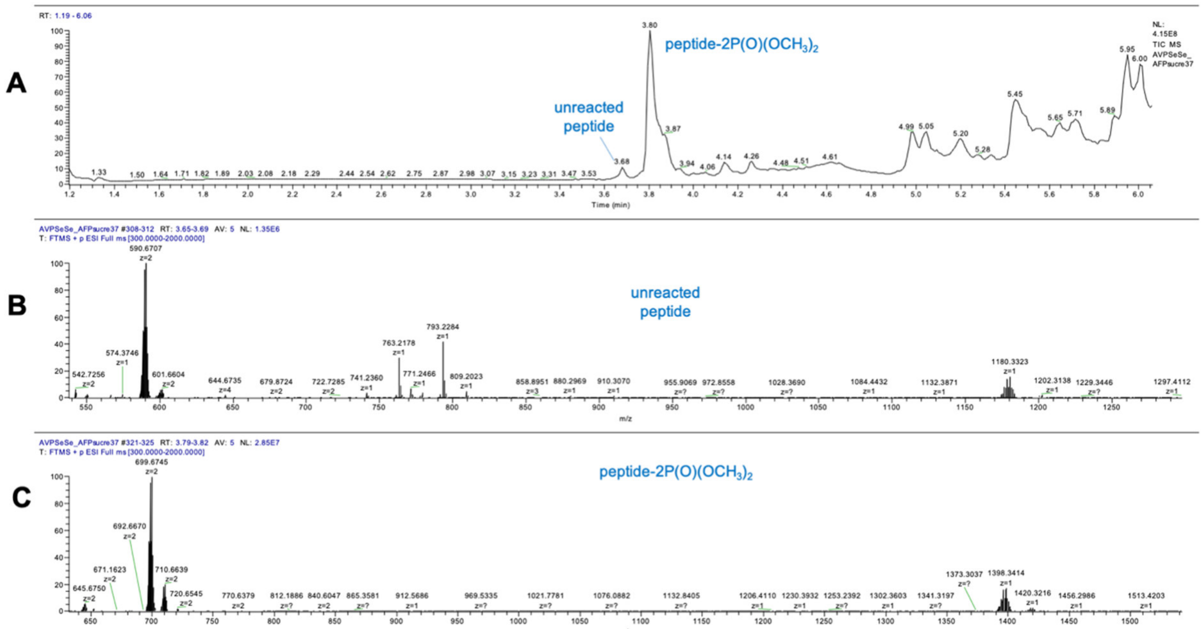

Figure S7. LC-MS of (Se-Se)-AVP incubated 18 h with [Au{P(OMe)$_3$}STga] (3 eq.) at 37 °C in presence of DTT. (**A**) TIC. (**B**) Mass spectrum of peak at t_R = 3.68 min. (**C**) Mass spectrum of peak at t_R = 3.80 min.

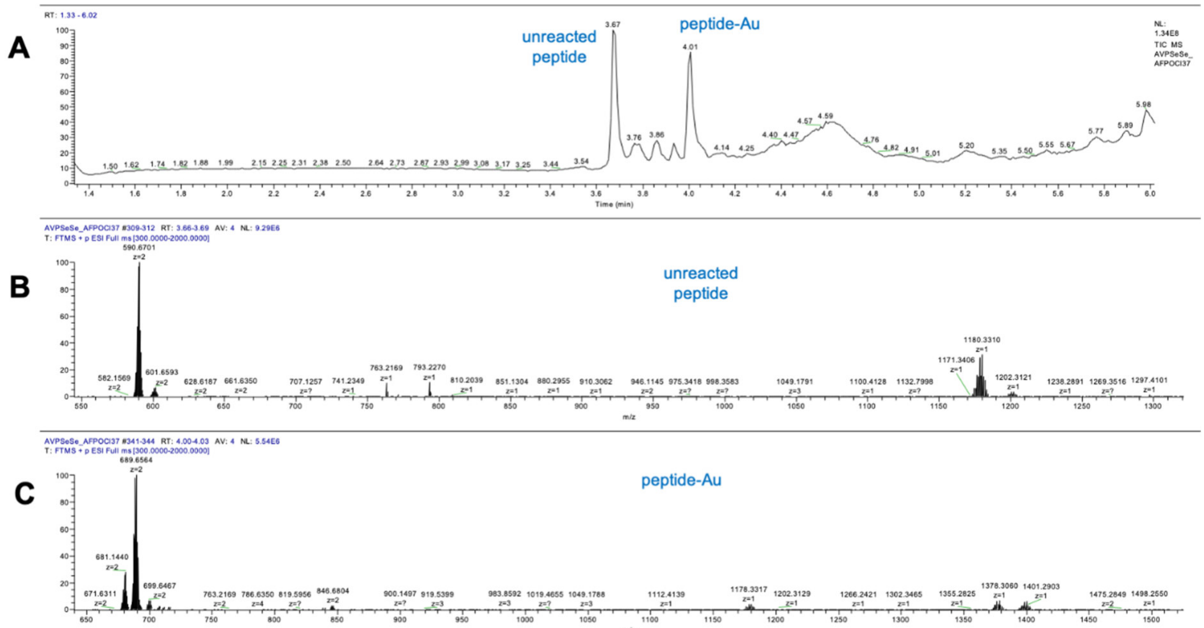

Figure S8. LC-MS of (Se-Se)-AVP incubated 18 h with [AuCl{P(OMe)$_3$}] (3 eq.) at 37 °C in presence of DTT. (**A**) TIC. (**B**) Mass spectrum of peak at t_R = 3.68 min. (**C**) Mass spectrum of peak at t_R = 4.00 min.

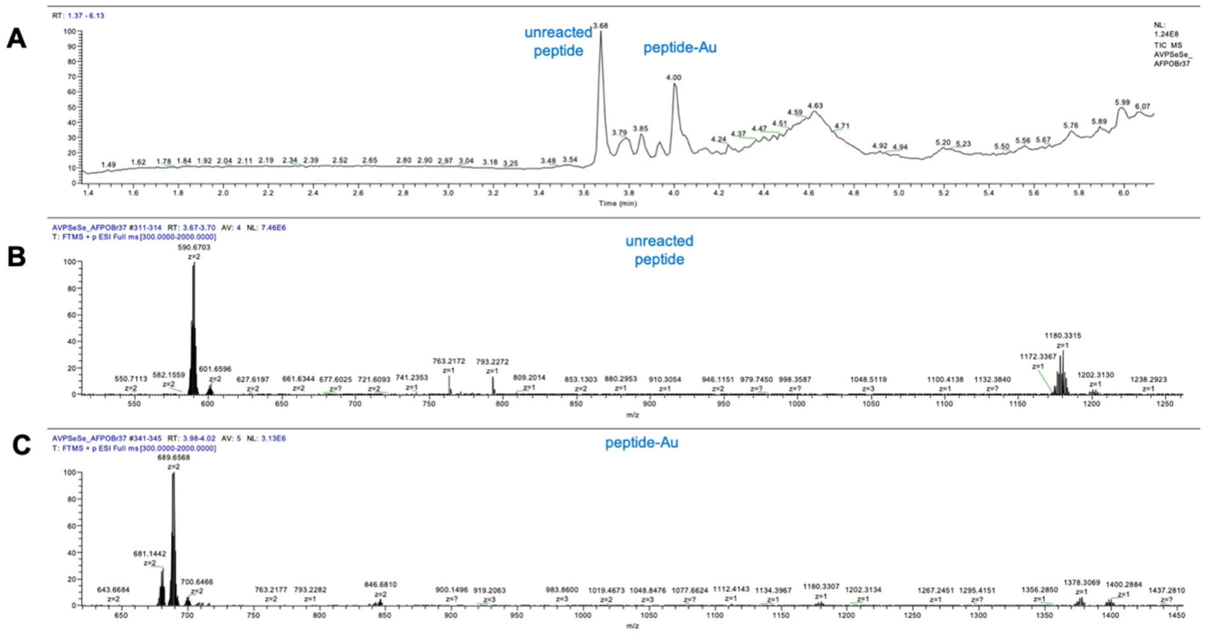

Figure S9. LC-MS of (Se-Se)-AVP incubated 18 h with [AuBr{P(OMe)$_3$}] (3 eq.) at 37 °C in presence of DTT. (**A**) TIC. (**B**) Mass spectrum of peak at t_R = 3.68 min. (**C**) Mass spectrum of peak at t_R = 4.00 min.

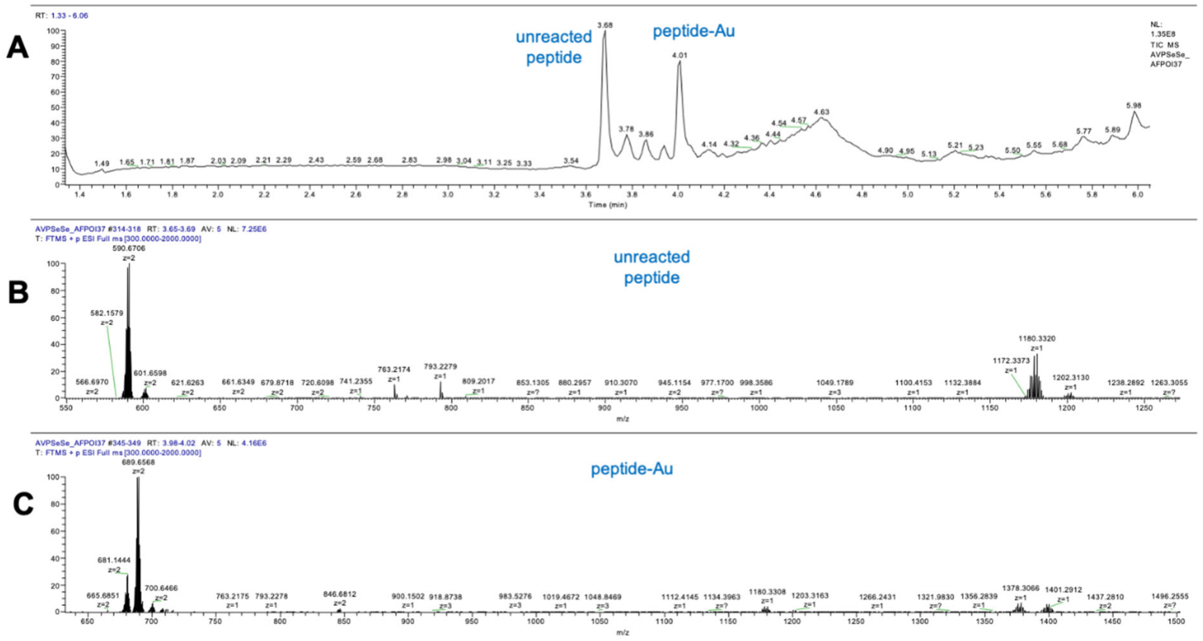

Figure S10. LC-MS of (Se-Se)-AVP incubated 18 h with [AuI{P(OMe)$_3$}] (3 eq.) at 37 °C in presence of DTT. (**A**) TIC. (**B**) Mass spectrum of peak at t_R = 3.68 min. (**C**) Mass spectrum of peak at t_R = 4.00 min.

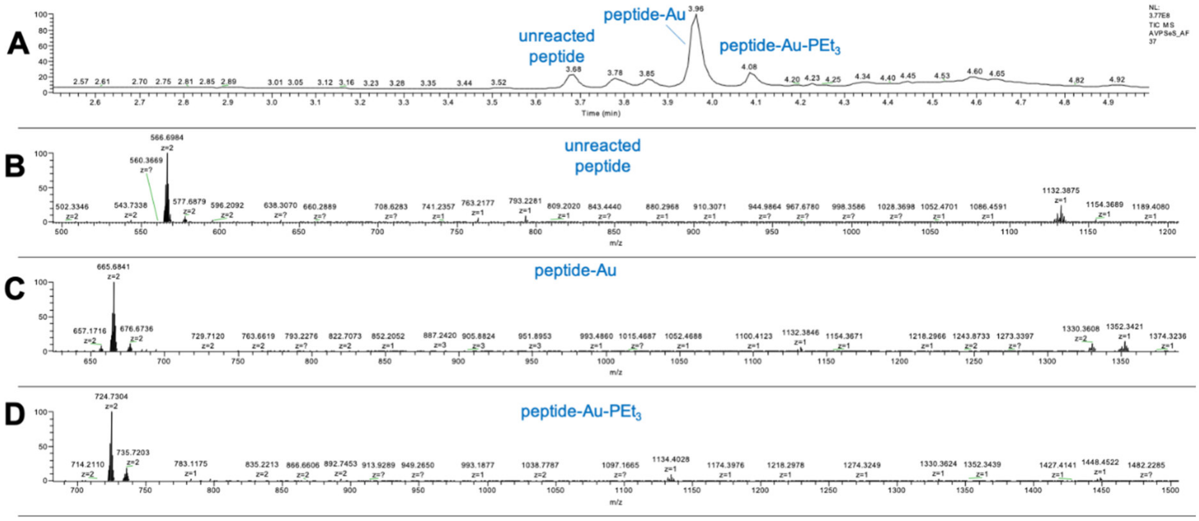

Figure S11. LC-MS of (S-Se)-AVP incubated 18 h with AF (3 eq.) at 37 °C in presence of DTT. (**A**) TIC. (**B**) Mass spectrum of peak at t_R = 3.68 min. (**C**) Mass spectrum of peak at t_R = 3.96 min. (**D**) Mass spectrum of peak at t_R = 4.08 min.

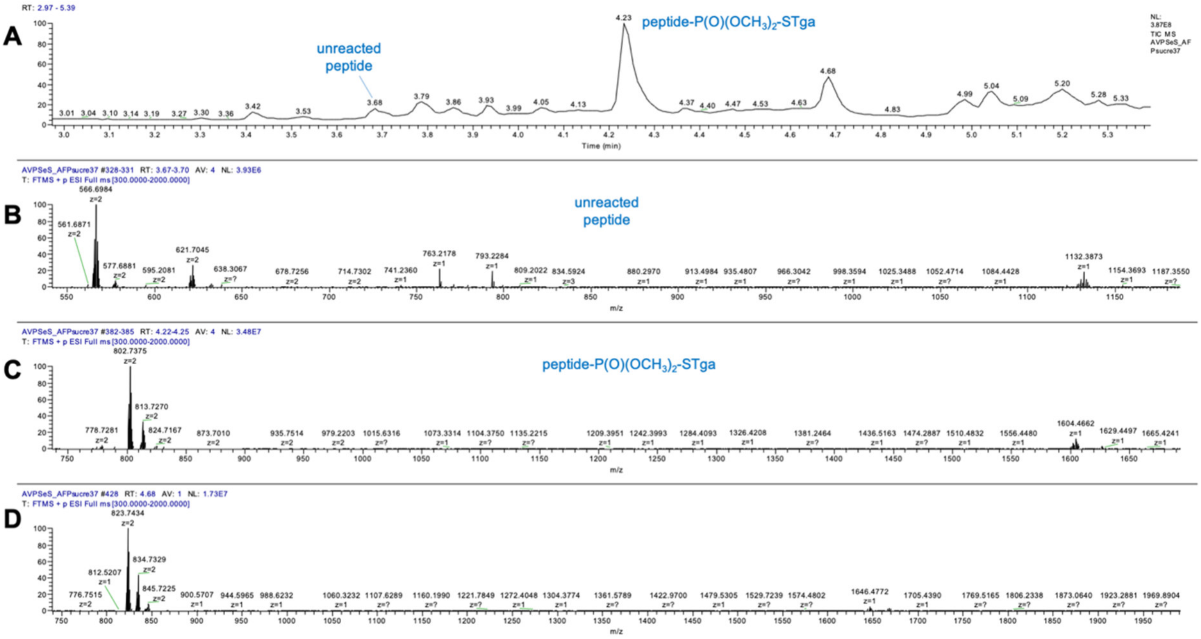

Figure S12. LC-MS of (S-Se)-AVP incubated 18 h with [Au{P(OMe)$_3$}STga] (3 eq.) at 37 °C in presence of DTT. (**A**) TIC. (**B**) Mass spectrum of peak at t_R = 3.68 min. (**C**) Mass spectrum of peak at t_R = 4.23 min.

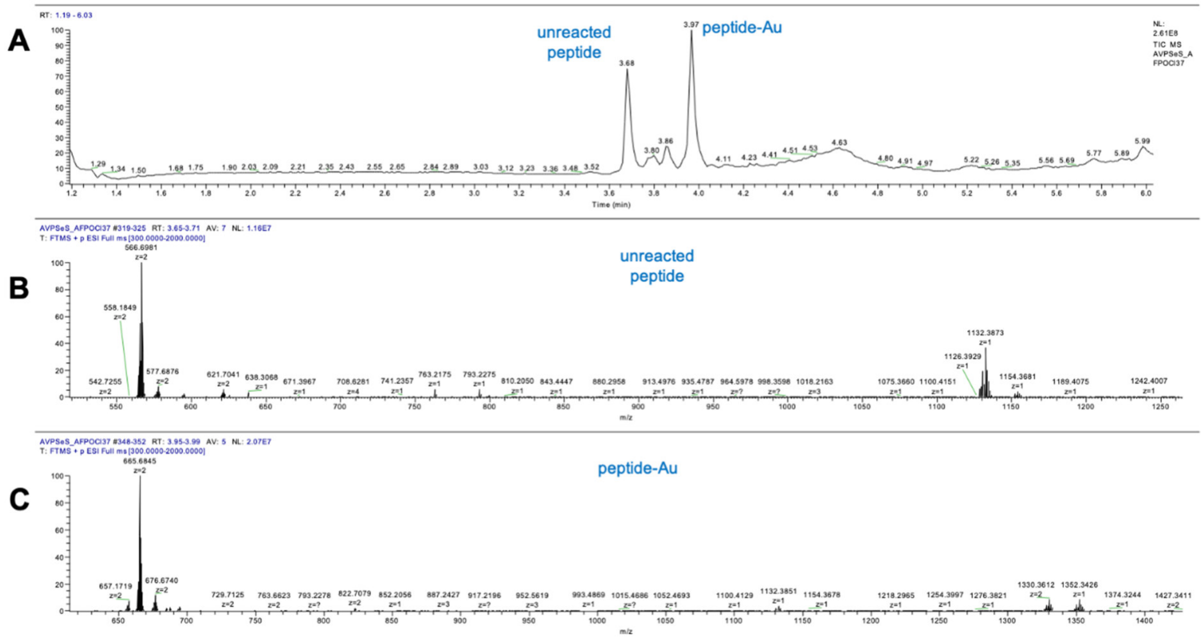

Figure S13. LC-MS of (S-Se)-AVP incubated 18 h with [AuCl{P(OMe)$_3$}] (3 eq.) at 37 °C in presence of DTT. (**A**) TIC. (**B**) Mass spectrum of peak at t_R = 3.68 min. (**C**) Mass spectrum of peak at t_R = 3.97 min.

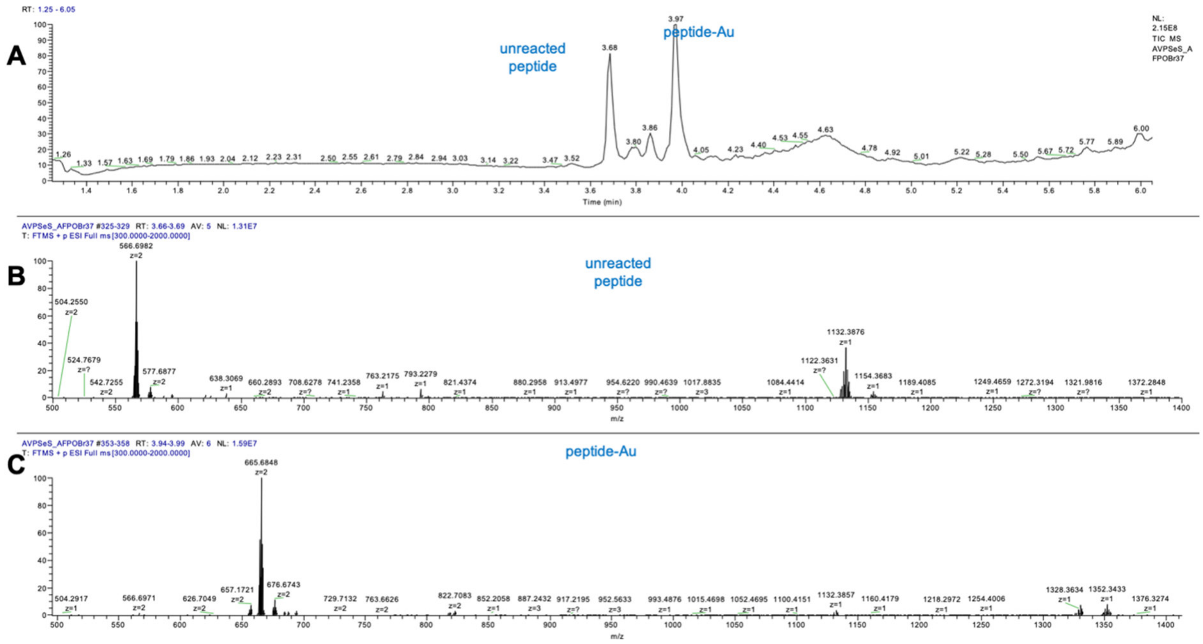

Figure S14. LC-MS of (S-Se)-AVP incubated 18 h with [AuBr{P(OMe)$_3$}] (3 eq.) at 37 °C in presence of DTT. (**A**) TIC. (**B**) Mass spectrum of peak at t_R = 3.68 min. (**C**) Mass spectrum of peak at t_R = 3.97 min.

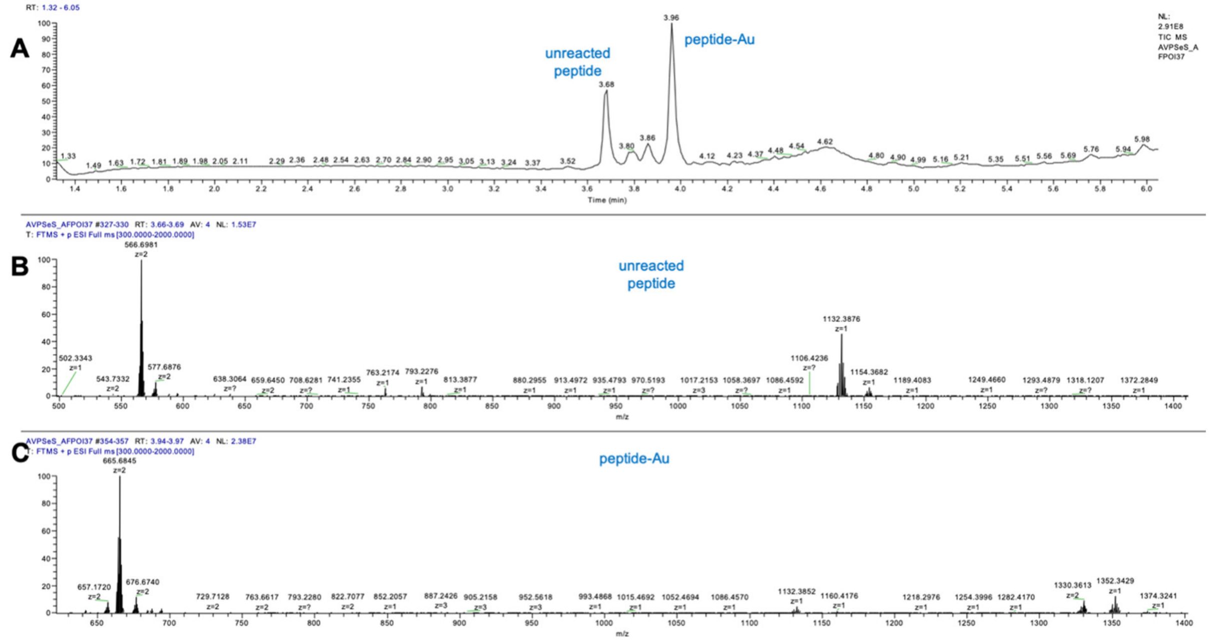

Figure S15. LC-MS of (S-Se)-AVP incubated 18 h with [AuI{P(OMe)$_3$}] (3 eq.) at 37 °C in presence of DTT. (**A**) TIC. (**B**) Mass spectrum of peak at t_R = 3.68 min. (**C**) Mass spectrum of peak at t_R = 3.97 min.

Triethylphosphine complexes ^{31}P NMR data

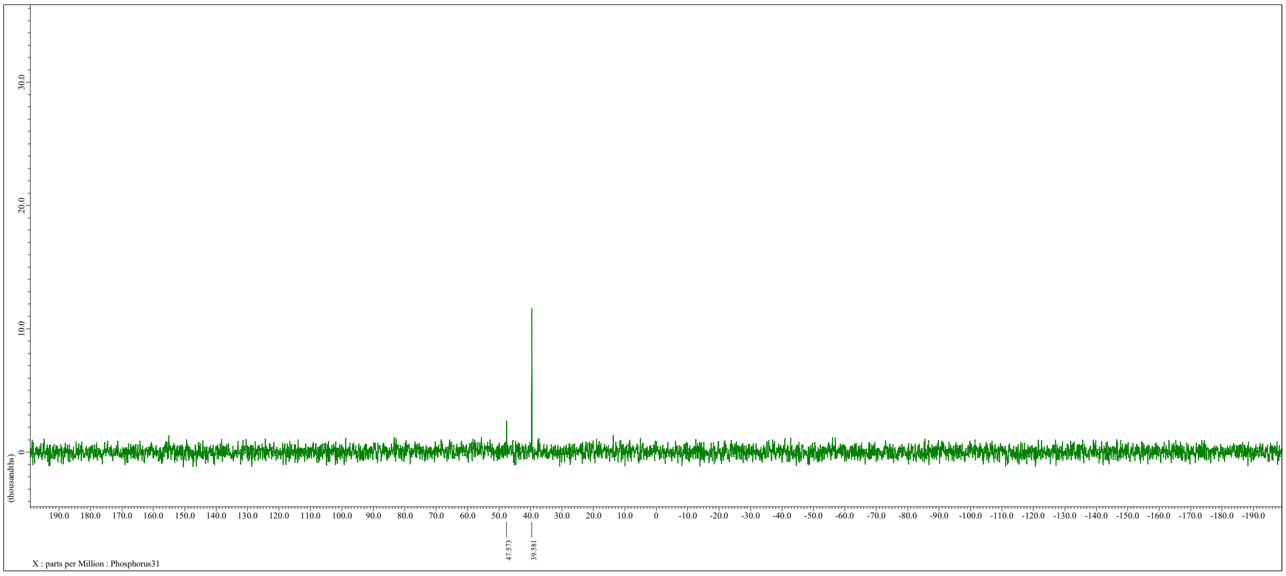

Figure S16. ^{31}PNMR spectrum of AF in presence of Cys recorded at t_0.

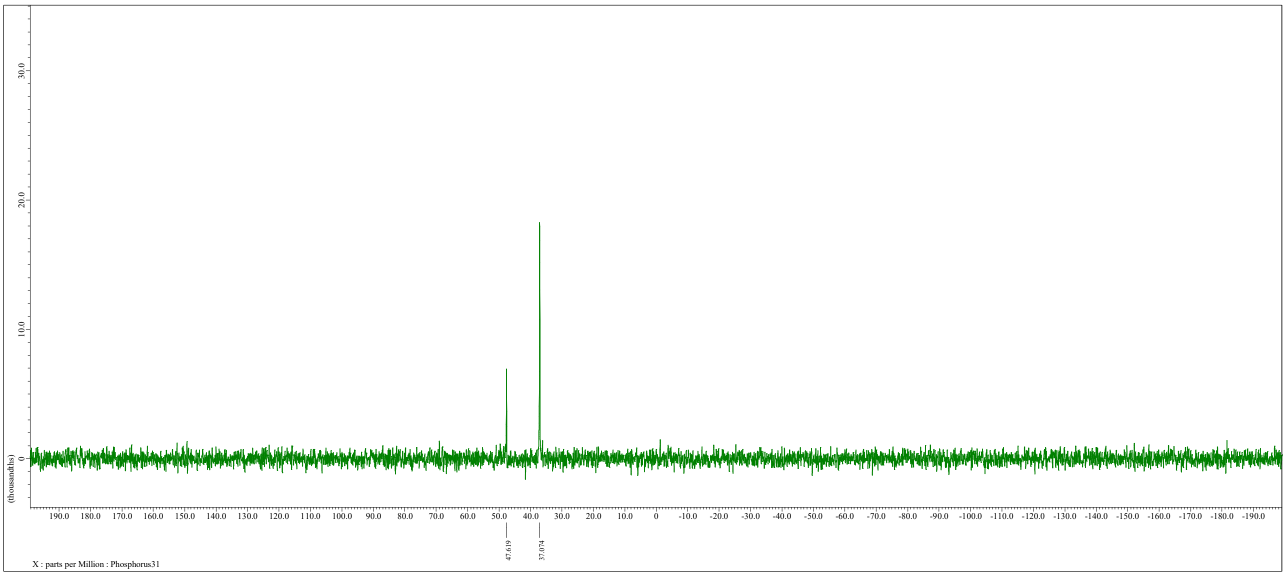

Figure S17. ^{31}PNMR spectrum of AF in presence of Cys recorded at 24h.

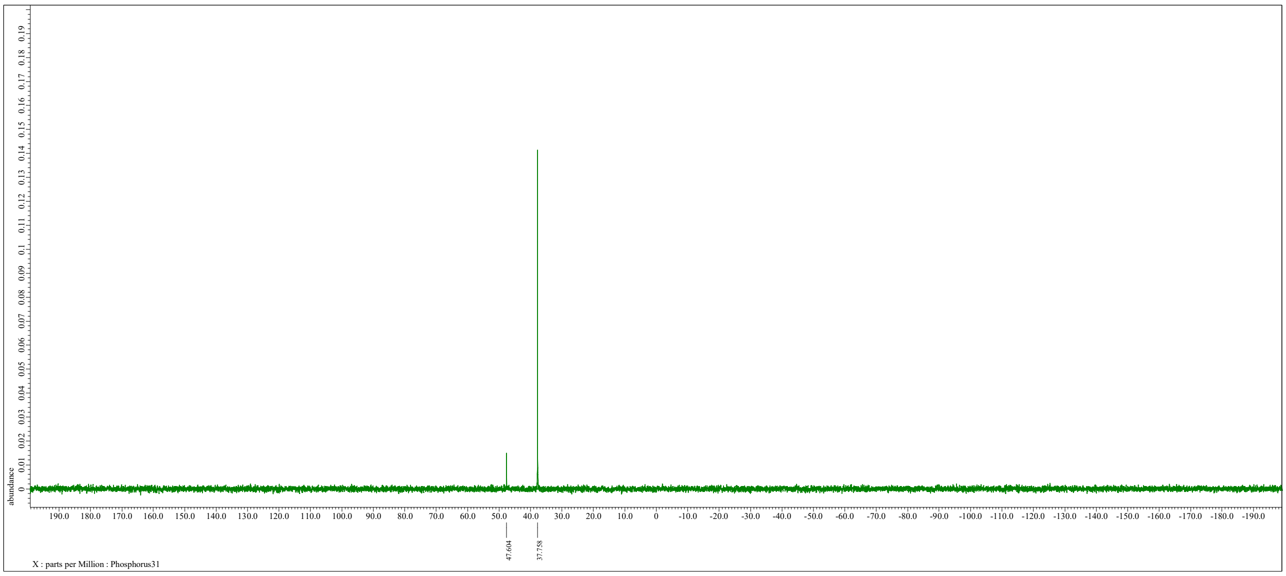

Figure S18. ^{31}PNMR spectrum of [AuCl(PEt_3)] in presence of Cys recorded at t_0.

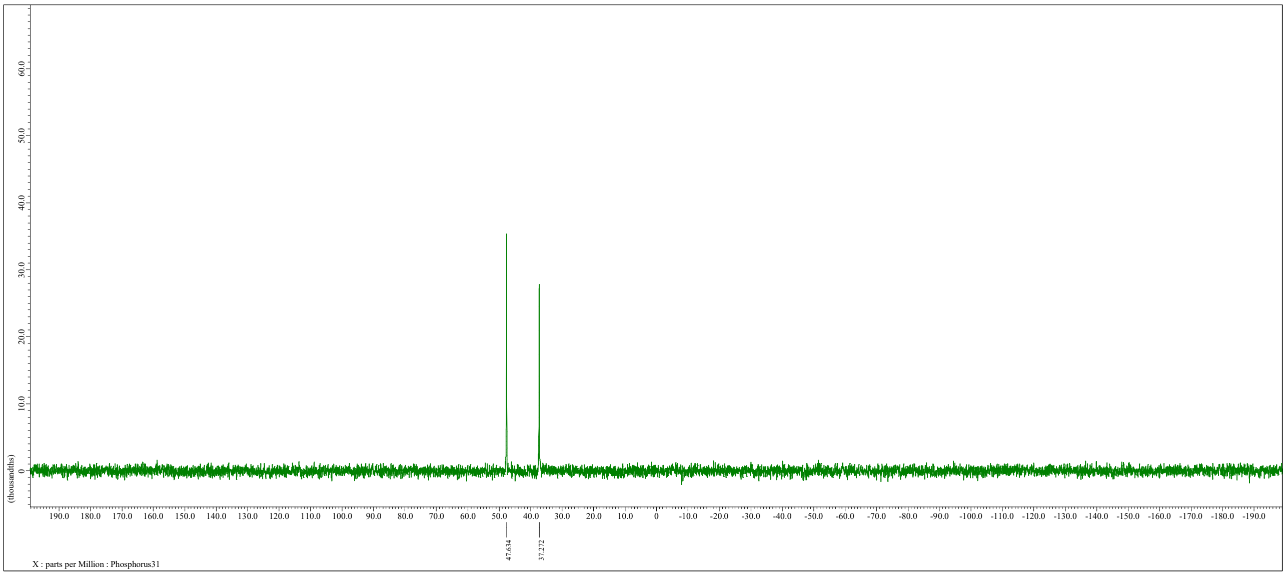

Figure S19. ^{31}PNMR spectrum of [AuCl(PEt_3)] in presence of Cys recorded at 24h.

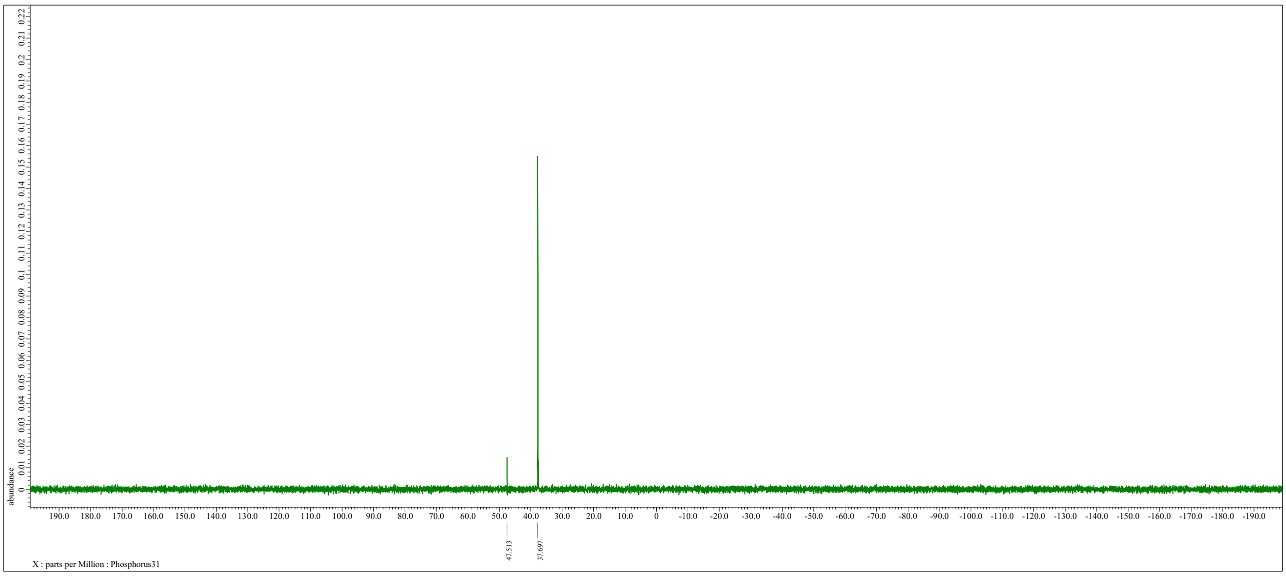

Figure S20. ^{31}PNMR spectrum of $[AuBr(PEt_3)]$ in presence of Cys recorded at t_0.

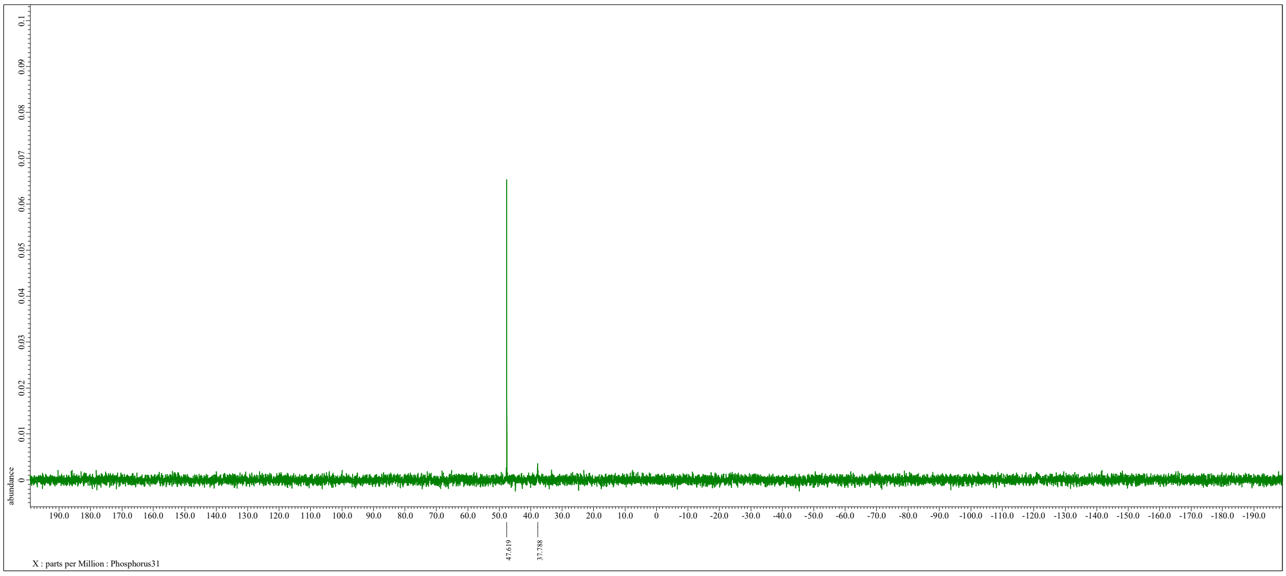

Figure S21. ^{31}PNMR spectrum of $[AuBr(PEt_3)]$ in presence of Cys recorded at 24h.

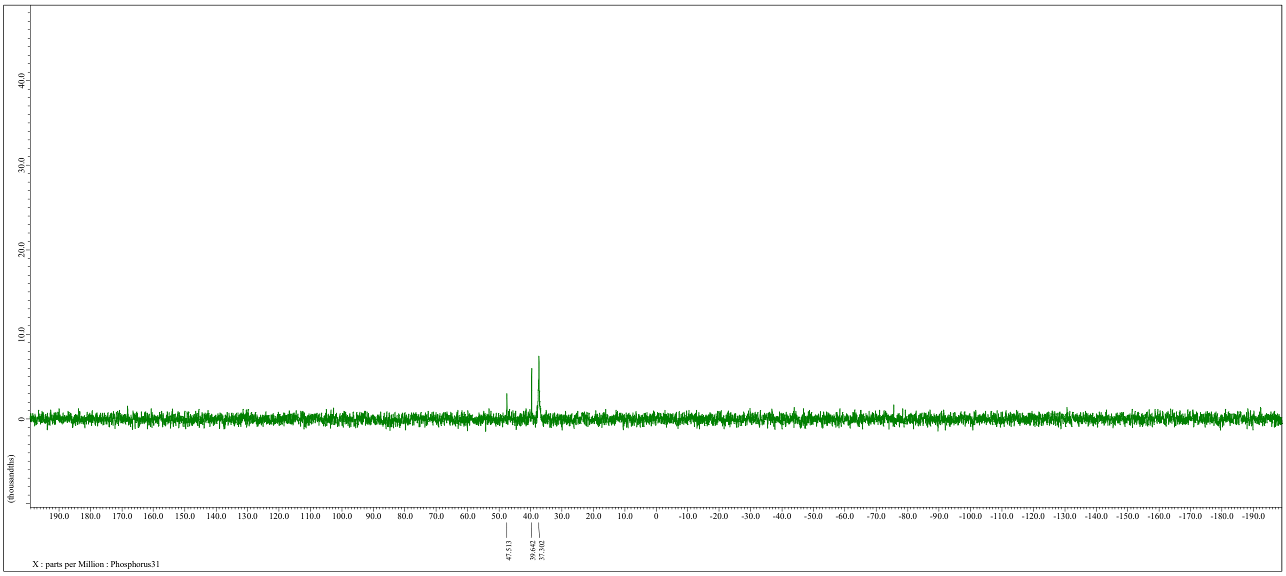

Figure S22. ^{31}PNMR spectrum of [AuI(PEt$_3$)] in presence of Cys recorded at t_0.

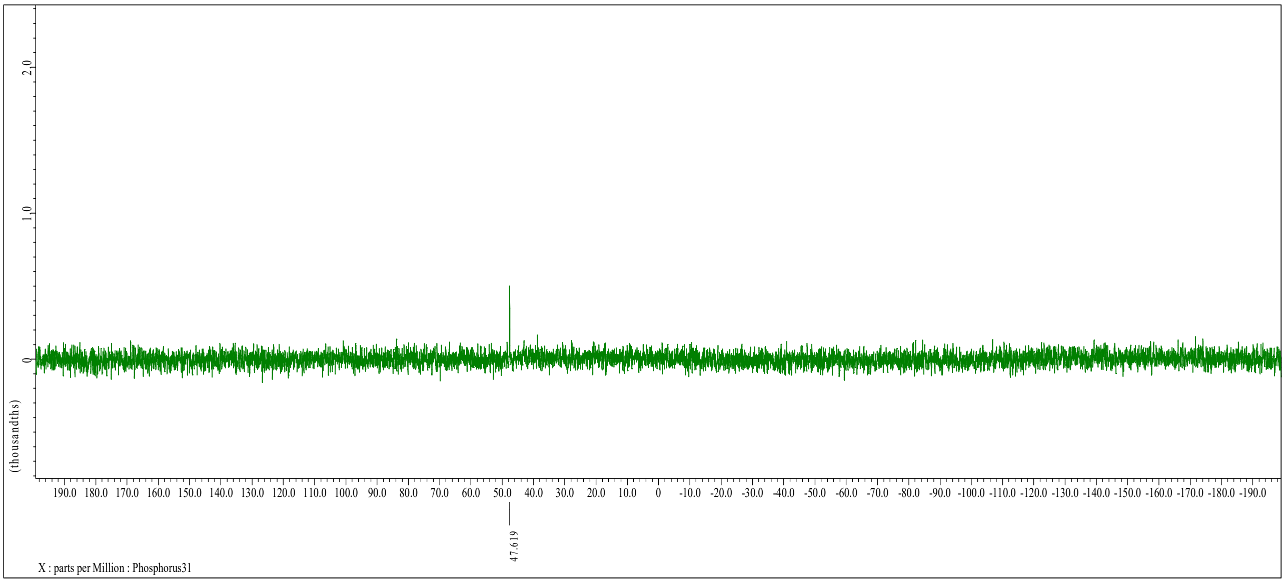

Figure S23. ^{31}PNMR spectrum of [AuI(PEt$_3$)] in presence of Cys recorded at 24h.

Trimethylphosphite complexes ^{31}P NMR data

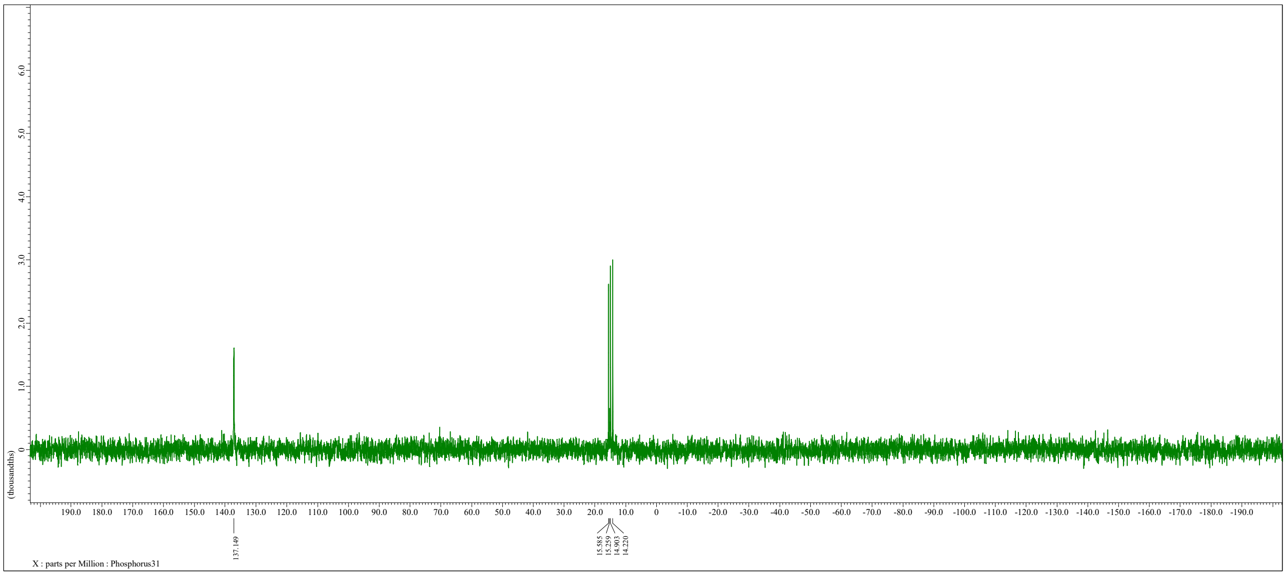

Figure S24. ^{31}PNMR spectrum of [Au{P(OMe)$_3$}STga] in presence of Cys recorded at t_0.

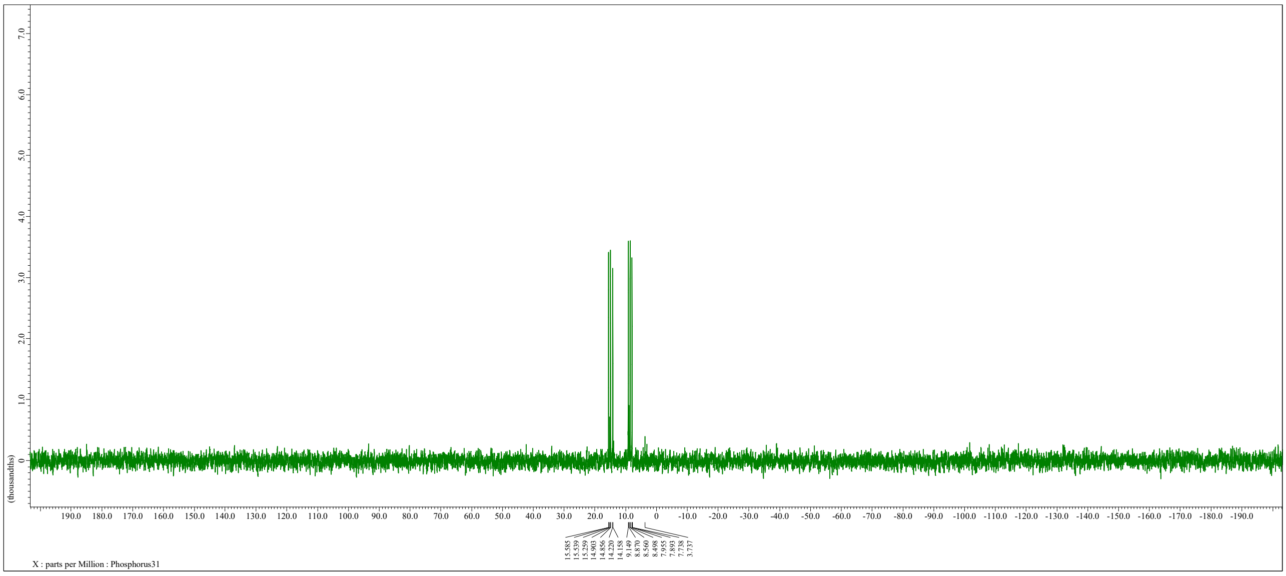

Figure S25. ^{31}PNMR spectrum of [Au{P(OMe)$_3$}STga] in presence of Cys recorded at 24h.

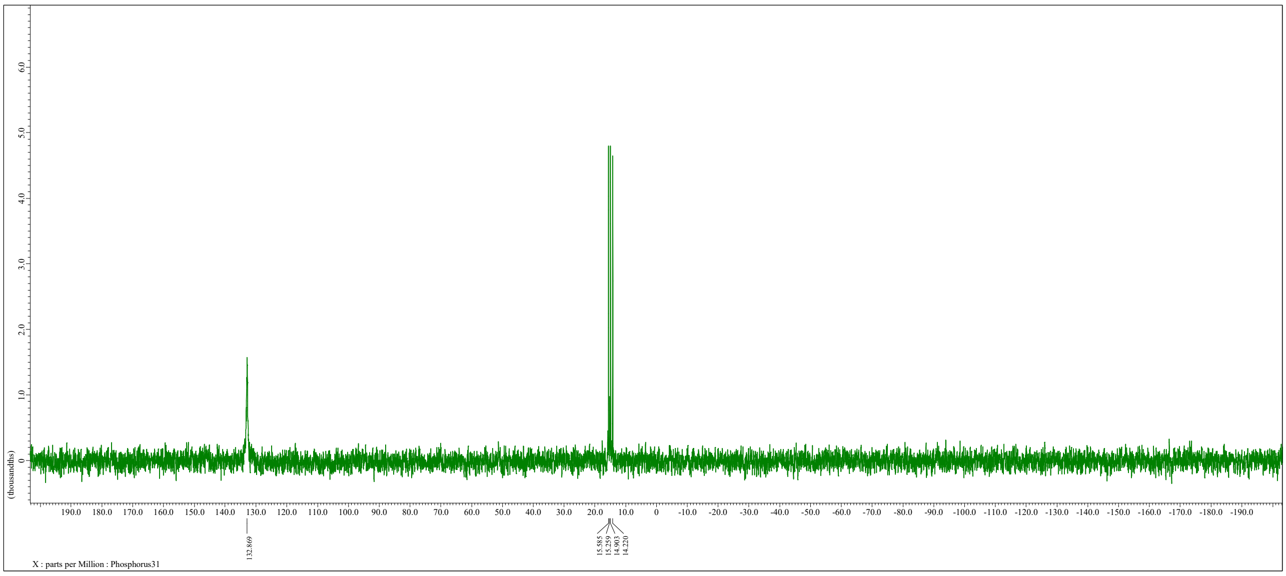

Figure S26. ^{31}P NMR spectrum [AuCl{P(OMe)$_3$}] in presence of Cys recorded at t_0.

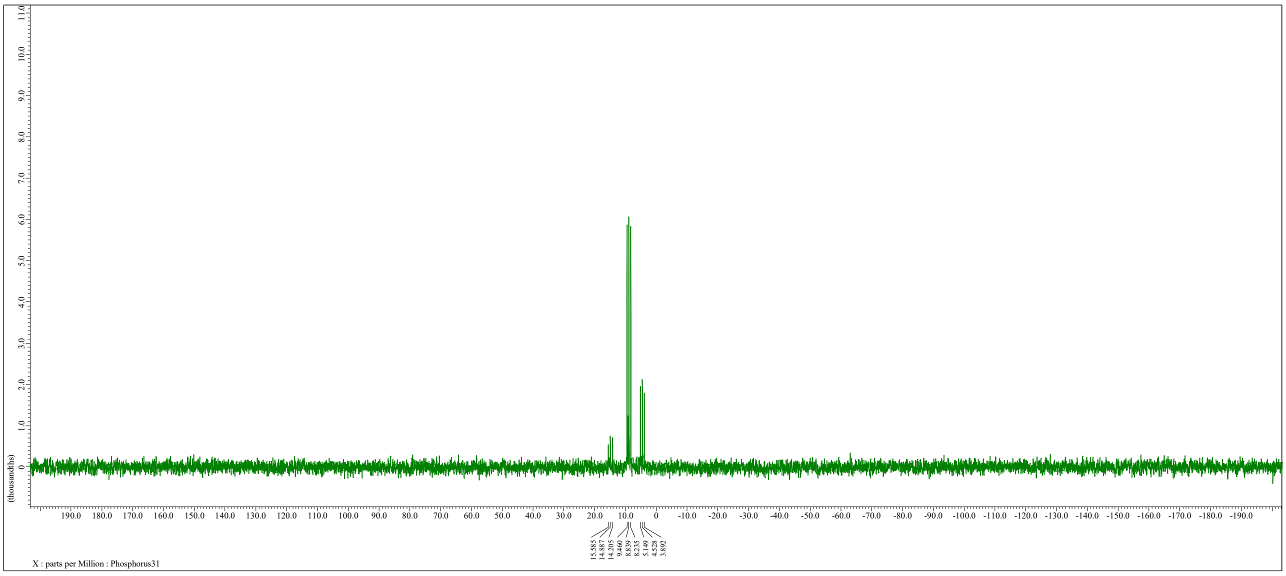

Figure S27. ^{31}P NMR spectrum of [AuCl{P(OMe)$_3$}] in presence of Cys recorded at 24h.

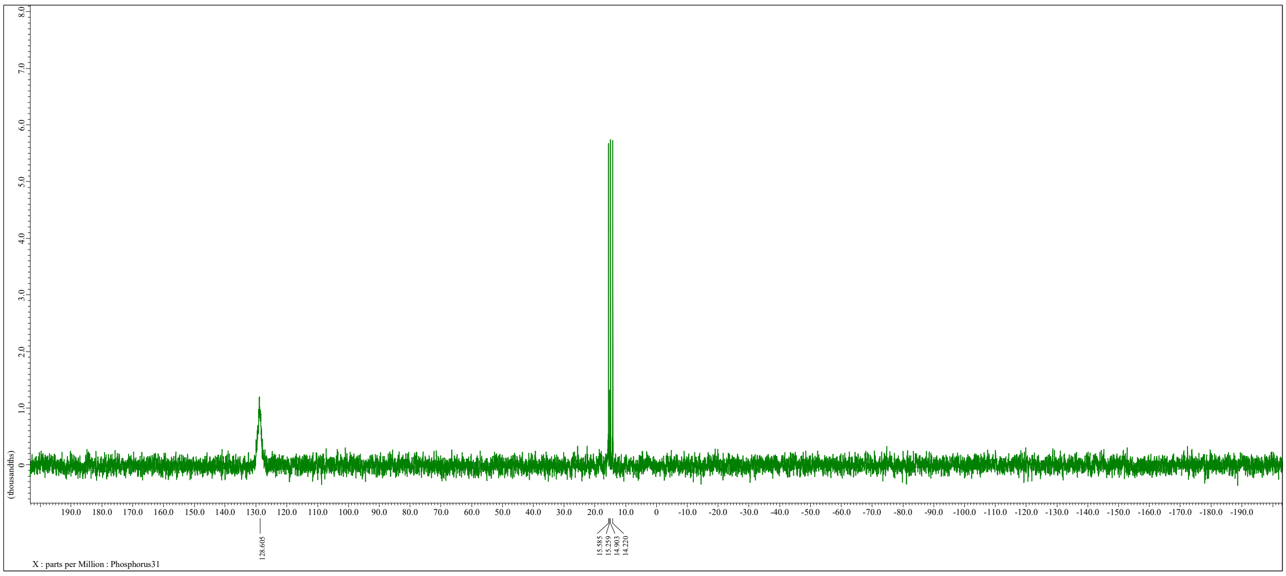

Figure S28. ^{31}P NMR spectrum of $[AuBr\{P(OMe)_3\}]$ in presence of Cys recorded at t_0.

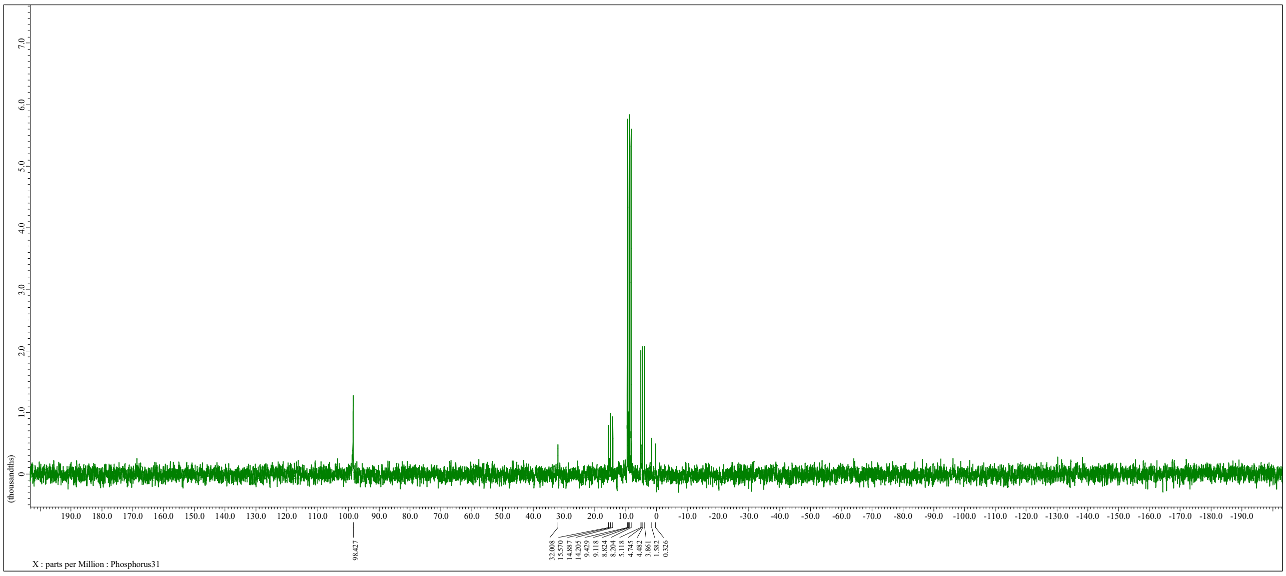

Figure S29. ^{31}P NMR spectrum of $[AuBr\{P(OMe)_3\}]$ in presence of Cys recorded at 24h.

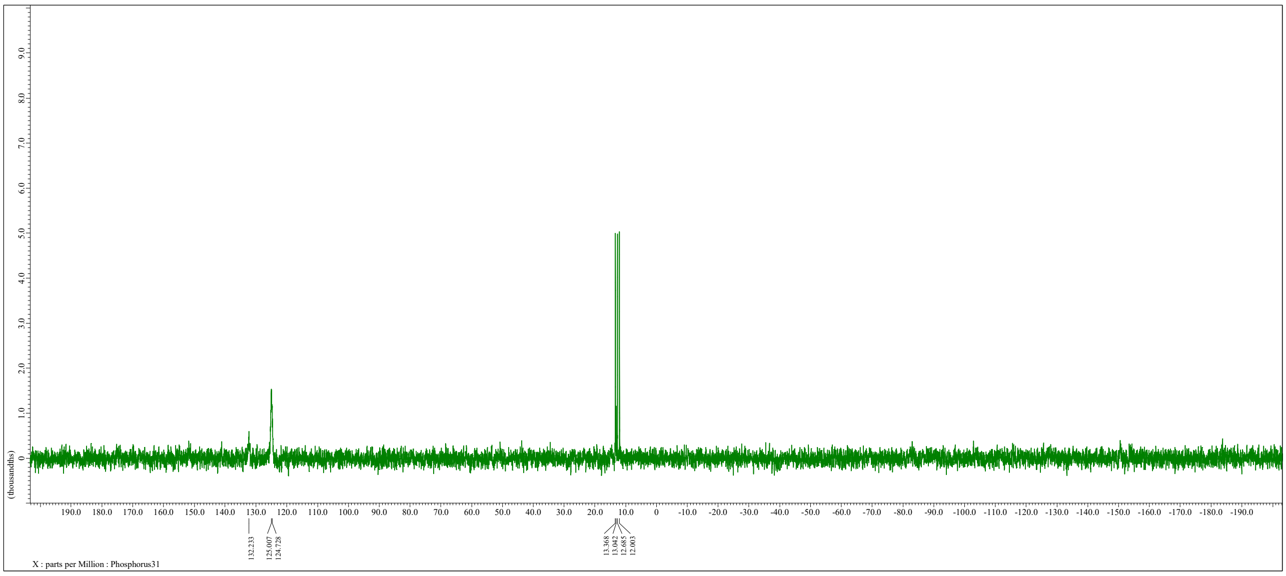

Figure S30. ^{31}P NMR spectrum of [AuI{P(OMe)$_3$}] in presence of Cys recorded at t_0.

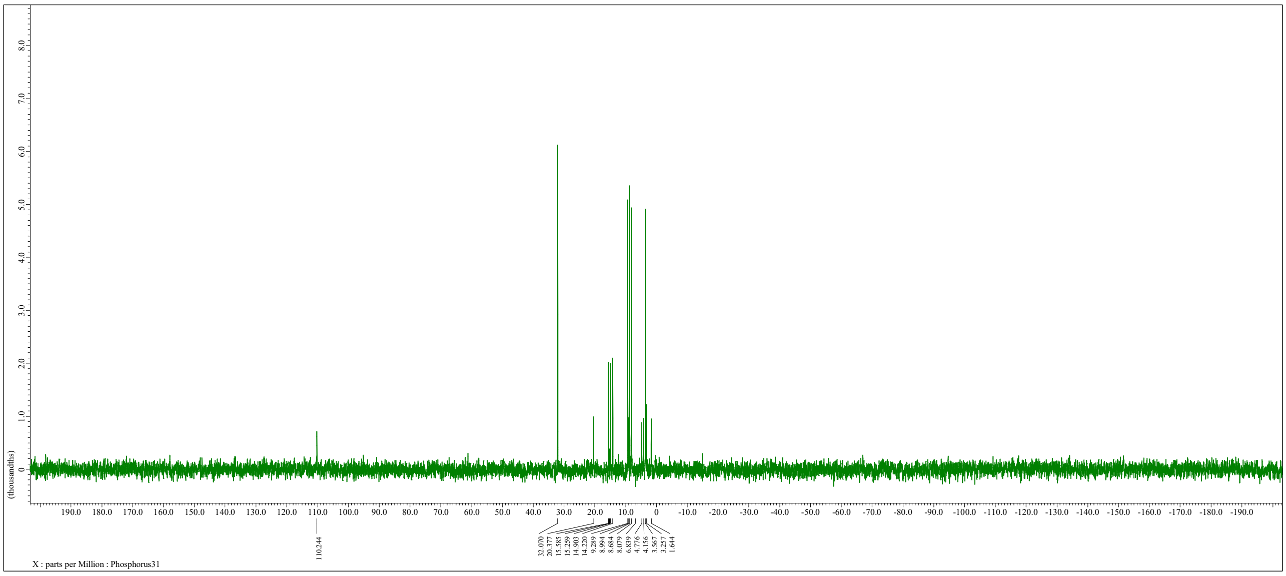

Figure S31. ^{31}PNMR spectrum of [AuI{P(OMe)$_3$}] in presence of Cys recorded at 24h.

Trimethylphosphite ^{31}P NMR spectrum

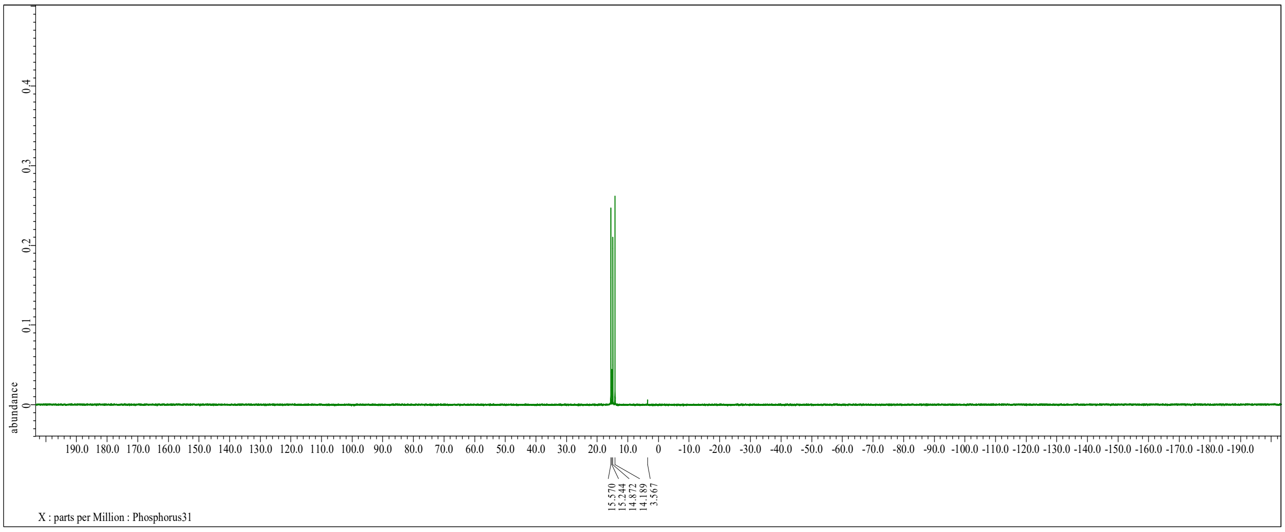

Figure S32. ^{31}P NMR spectrum of thimethylphosphite.
